# The composition alteration of gut microbiota in lung cancer: a systematic review and meta-analysis

**DOI:** 10.3389/fmicb.2026.1873706

**Published:** 2026-07-20

**Authors:** Xiaona Xu, Ting Yu, Hongying Wu, Yang Guo, Meng Li, Yaozu Han, Lei Zhao, Xinjuan Yu

**Affiliations:** 1 Central Laboratory, Qingdao Municipal Hospital, University of Health and Rehabilitation Sciences, Qingdao, China; 2 Qingdao Traditional Chinese Medicine Hospital, Qingdao Hiser Hospital Affiliated of Qingdao University, Qingdao, China; 3 School of Medical Laboratory, Shandong Second Medical University, Weifang, China; 4 Qingdao Central Hospital, University of Health and Rehabilitation Sciences, Qingdao, China; 5 Clinical Research Center, Qingdao Key Laboratory of Common Diseases, Qingdao Municipal Hospital, University of Health and Rehabilitation Sciences, Qingdao, China; 6 Human Resources Department, Qingdao Municipal Hospital, University of Health and Rehabilitation Sciences, Qingdao, China

**Keywords:** dysbiosis, gut microbiota, lung cancer, meta-analysis, metagenome, systematic review

## Abstract

**Background:**

The association between the gut microbiota and lung cancer remains understudied. In this study, we conducted a comprehensive systematic review and meta-analysis to quantitatively synthesize evidence from multiple cohorts to identify robust and consistent alterations in gut microbial diversity and taxonomy associated with lung cancer.

**Methods:**

A systematic literature search was performed across PubMed, Cochrane Library, Embase, and Web of Science databases up to June 5, 2025. The analysis summarized key microbiota characteristics from the selected studies, including alpha diversity, beta diversity, and relative taxonomic abundance. This meta-analysis was conducted in accordance with the Preferred Reporting Items for Systematic Reviews and Meta-Analyses (PRISMA) 2020 guidelines.

**Results:**

Our systematic search identified 12,810 articles, out of which 27 studies comprising 2,263 individuals, involving 1,234 lung cancer patients and 1,029 non-cancer controls, were included for qualitative synthesis. Meta-analysis revealed a significant reduction in microbial alpha diversity of 25 studies in lung cancer patients. Significant decreases were indicated in the ACE index (SMD = −0.64, 95% CI: −1.14 to −0.13), Chao1 index (SMD = −0.31, 95% CI: −0.60 to −0.02), and Shannon index (SMD = −0.25, 95% CI: −0.57 to 0.08). Chinese cohorts showed significantly lower Chao1and Shannon by subgroup analysis. Twenty-seven studies assessed beta diversity, in which 20 studies (74.0%) reported a significant difference in overall microbial community structure between lung cancer patients and non-cancer controls. Quantitative meta-analysis by forest plot revealed, compared to non-cancer controls, lung cancer patients exhibited decreased relative abundances of phylum Firmicutes (SMD = −0.47, 95% CI: −0.91 to −0.02), and increased abundances of phylum Bacteroidetes (SMD = 0.53, 95% CI: 0.24 to 0.82). Furthermore, we observed a marked depletion of beneficial short-chain fatty acid producers of genus *Lachnospira* (SMD = −1.01, 95% CI: −1.29 to −0.73).

**Conclusion:**

This meta-analysis demonstrates that lung cancer is consistently associated with gut microbiota dysbiosis characterized by reduced microbial diversity and reproducible taxonomic alterations. Clinically, these findings suggest that gut microbiota may serve as non-invasive biomarkers for lung cancer detection and patient stratification, and may also help predict immunotherapy response and inform future microbiota-targeted therapeutic strategies.

**Systematic review registration:**

https://www.crd.york.ac.uk/PROSPERO/view/CRD42024537463, CRD42024537463.

## 1 Introduction

Lung cancer remains the leading cause of cancer-related mortality worldwide, accounting for more than 1.8 million deaths annually (Adjei, 2019; Huang et al., 2022; Leiter et al., 2023). Non-small cell lung cancer (NSCLC), which constitutes approximately 85% of all lung cancer cases, is often diagnosed at advanced stages, contributing to its persistently poor prognosis (Rudin et al., 2021; Chen P. et al., 2022; Hendriks et al., 2024). Despite recent breakthroughs in targeted therapies and immunotherapies, including immune checkpoint inhibitors (ICIs), clinical response remains heterogeneous, and predictive biomarkers for early detection and treatment efficacy remain insufficient (Mountzios et al., 2023; Nabet et al., 2024). As the complexity of lung cancer pathogenesis becomes increasingly apparent, attention has turned toward novel systemic influences, such as the human microbiome (Sepich-Poore et al., 2021; Liu et al., 2024).

The human microbiota, comprising trillions of microorganisms inhabiting distinct anatomical niches, is now recognized as a pivotal modulator of host immunity, metabolism, and inflammatory tone (Wang et al., 2017; Rackaityte and Lynch, 2020). Of particular interest is the gut microbiota, a highly dynamic ecosystem of microorganisms that interact intricately with host metabolism, immune surveillance, and epithelial integrity (Fan and Pedersen, 2021; Suzuki et al., 2022). Dysbiosis, a disruption in microbial diversity and stability, has been implicated in several malignancies, including colorectal, pancreatic, and hepatocellular carcinomas (Yang et al., 2021; Hsu and Schnabl, 2023). Increasing evidence suggests that gut microbial dysbiosis in the etiology and progression of lung cancer may be mediated through the gut-lung axis, a bidirectional communication network in which gut microbes and their metabolites shape pulmonary immune tone, systemic inflammation, and antitumor immune responses (Zhuang et al., 2019; Qin et al., 2022). Microbiota-derived products, particularly short-chain fatty acids and other immunomodulatory metabolites, may influence lung carcinogenesis by regulating epithelial barrier integrity, inflammatory signaling, and the tumor immune microenvironment, thereby affecting both disease development and treatment responsiveness (Liu et al., 2020; Li et al., 2024).

Multiple studies have demonstrated significant compositional and functional differences in the gut microbiota of lung cancer patients compared to healthy controls. Taxonomic shifts often include the enrichment of pro-inflammatory or opportunistic genera such as *Streptococcus*, *Escherichia*, and *Enterococcus*, alongside a depletion of beneficial commensals like *Bifidobacterium*, *Faecalibacterium*, and *Blautia* (Jiang et al., 2023; Ni et al., 2023). Moreover, emerging clinical studies have linked specific gut microbial signatures with lung cancer treatment outcomes. For example, *Bifidobacterium breve* abundance has been associated with improved response to anti-PD-1 therapy when combined with chemotherapy (Zhao et al., 2023), while *Akkermansia muciniphila* has been implicated in enhancing the efficacy of immune checkpoint blockade in other cancers and may play a similar role in lung malignancies (Haberman et al., 2023). These findings support the notion that the gut microbiota modulates systemic antitumor immunity, influencing not only cancer risk but also therapeutic responsiveness (Mager et al., 2020; Lu et al., 2022). However, the precise mechanisms by which gut microbiota exert influence over the lung tumor microenvironment remain incompletely understood.

Despite this growing body of literature, our understanding of gut microbiota alterations in lung cancer remains fragmented. Existing studies have largely examined the gut microbiomes in isolation, often with modest sample sizes and variable methodologies. In this study, we performed a comprehensive meta-analysis encompassing gut microbiota in lung cancer patients across multiple clinical cohorts. The primary research question was whether lung cancer is associated with reproducible alterations in gut microbial diversity and taxonomic composition compared with non-cancer controls. We hypothesized that patients with lung cancer would exhibit a consistent pattern of gut microbiota dysbiosis, characterized by reduced alpha diversity and distinct taxonomic shifts. Our analysis reveals consistent patterns of microbial dysbiosis across gut compartments, and underscores the systemic nature of microbe-host-tumor interactions. This work not only enhances our understanding of the microbiome’s role in lung cancer but also provides a foundational framework for microbiota-informed strategies in early detection, patient stratification, and treatment personalization.

## 2 Materials and methods

### 2.1 Protocol and registration

The protocol for the meta-review has been officially logged with PROSPERO (registration number: CRD42024537463), and searches were conducted in accordance with the updated 2020 Preferred Reporting Items for Systematic Reviews and Meta-Analyses (PRISMA)statement (Page et al., 2021) and checklist. The PROSPERO link is https://www.crd.york.ac.uk/PROSPERO/view/CRD42024537463.

The objective of this review assessment was to analyze and compare the variations in the diversity patterns of gut microbiota, along with the relative abundance of distinct bacterial phyla, families, and genera between lung cancer patients and healthy controls or benign lung nodule controls.

### 2.2 Literature source and search strategy

On June 5, 2025, the systematic literature search was conducted using four databases (PubMed®, Cochrane Library, Embase®, and Web of Science™) by two authors independently. The search strategy in PubMed was: (((((microbiota[MeSH Terms]) OR (microbiota[Title/Abstract] OR microbiome*[Title/Abstract] OR microbiota*[Title/Abstract] OR microbial communit*[Title/Abstract])) OR (flore*[Title/Abstract] OR flora[Title/Abstract] OR microflor*[Title/Abstract])) OR ((metagenome[MeSH Terms]) OR (metagenome[Title/Abstract] OR metagenomes[Title/Abstract]))) OR ((dysbiosis[MeSH Terms]) OR (dysbiosis[Title/Abstract] OR dysbioses[Title/Abstract] OR disbiosis[Title/Abstract] OR disbioses[Title/Abstract] OR dysbacterios*[Title/Abstract] OR disbacterios*[Title/Abstract] OR bacterial[Title/Abstract]))) AND ((lung neoplasms[MeSH Terms]) OR (lung cancer*[Title/Abstract] OR lung neoplasms[Title/Abstract] OR lung neoplasm[Title/Abstract] OR pulmonary neoplasm*[Title/Abstract] OR pulmonary cancer*[Title/Abstract] OR Cancer of the Lung[Title/Abstract] OR Cancer of Lung[Title/Abstract])). The literature search strategy was appropriated for Cochrane Library, Embase®, and Web of Science™ databases. We also searched for articles in the references to avoid missing relevant research. The database search equations was provided in thef Supplementary Table 1.

### 2.3 Inclusion and exclusion criteria

Titles and abstracts were independently screened by two investigators. Any divergences between the two investigators were resolved through discussion with a third investigators. First, articles underwent a preliminary screening process based on their titles and abstracts, which was subsequently followed by a rigorous full-text review to ascertain eligibility according to the inclusion criteria. The ultimate selection was achieved via a consensus agreement among all contributing authors. The three investigators unanimously conducted literature screening according to the following inclusion and exclusion criteria.

Studies that fulfilled the following specified criteria were incorporated into the research: (1) the research design is an observational case–control study or a cross-sectional study; (2) there is at least one case group comprising individuals diagnosed with lung cancer, as well as at least one control group includes healthy subjects or patients with benign pulmonary nodules; (3) metagenomic sequencing or 16S rRNA sequencing analysis was implemented; (4) they reported the gut microbiota characteristics for both the control group and LC patients; (5) the study must describe the following outcome measures: at least one alpha diversity index (such as the number of operating taxonomic units (OTUs), Shannon index, Chao 1, Simpson index), and/or beta diversity, and/or the relative abundance of bacteria at the phylum, family, or genus level, in all groups; (6) the outcome indicators listed in (5) are provided in either the article or its Supplementary materials (including tables and figures), with specific numerical values that can be extracted from relevant charts; and (7) the language of their publication is English.

Studies that met any of the listed criteria were excluded from the study: (1) repeated publications or literature with similar data; (2) they reported on trials that were not carried out in humans; (3) types of literature such as abstracts, case reports, expert opinions, comments, letters, and editorials; (4) studies lacking a comparative qualified control group; (5) data on gut microbiota are not involved in the research; (6) the article involves other intervention factors; (7) the literature is not published in English; and (8) the study suffers from unclear data, an inability to extract information, or incorrect data.

### 2.4 Data extraction

Data were independently extracted from the included studies by two authors (Xu and Yu) and recorded in a self-designed data extraction table. To ensure consistent screening criteria, two investigators underwent standardized training prior to conducting the formal literature screening. The content of the extracted data comprises: (1) basic information (title, first author’s name, year of publication, location of the study (country/region), published journal, type of research design, sample type); (2) population characteristics (population source, sample size, age, gender); (3) sequencing information (sequencing platform, 16S rRNA amplification region); (4) research results (details of the gut microbiome, including alpha diversity index, beta diversity, and/or relative abundance of bacterial phyla, families, or genera). When the extraction of specific data from charts was required, quantitative measures of alpha diversity were procured utilizing the Get Data Graph Digitizer software. Extracted data were independently assessed by two reviewers. Any disagreements were resolved through discussion, and unresolved discrepancies were adjudicated by a third reviewer.

### 2.5 Quality assessment and risk of bias

**Figure 1:**
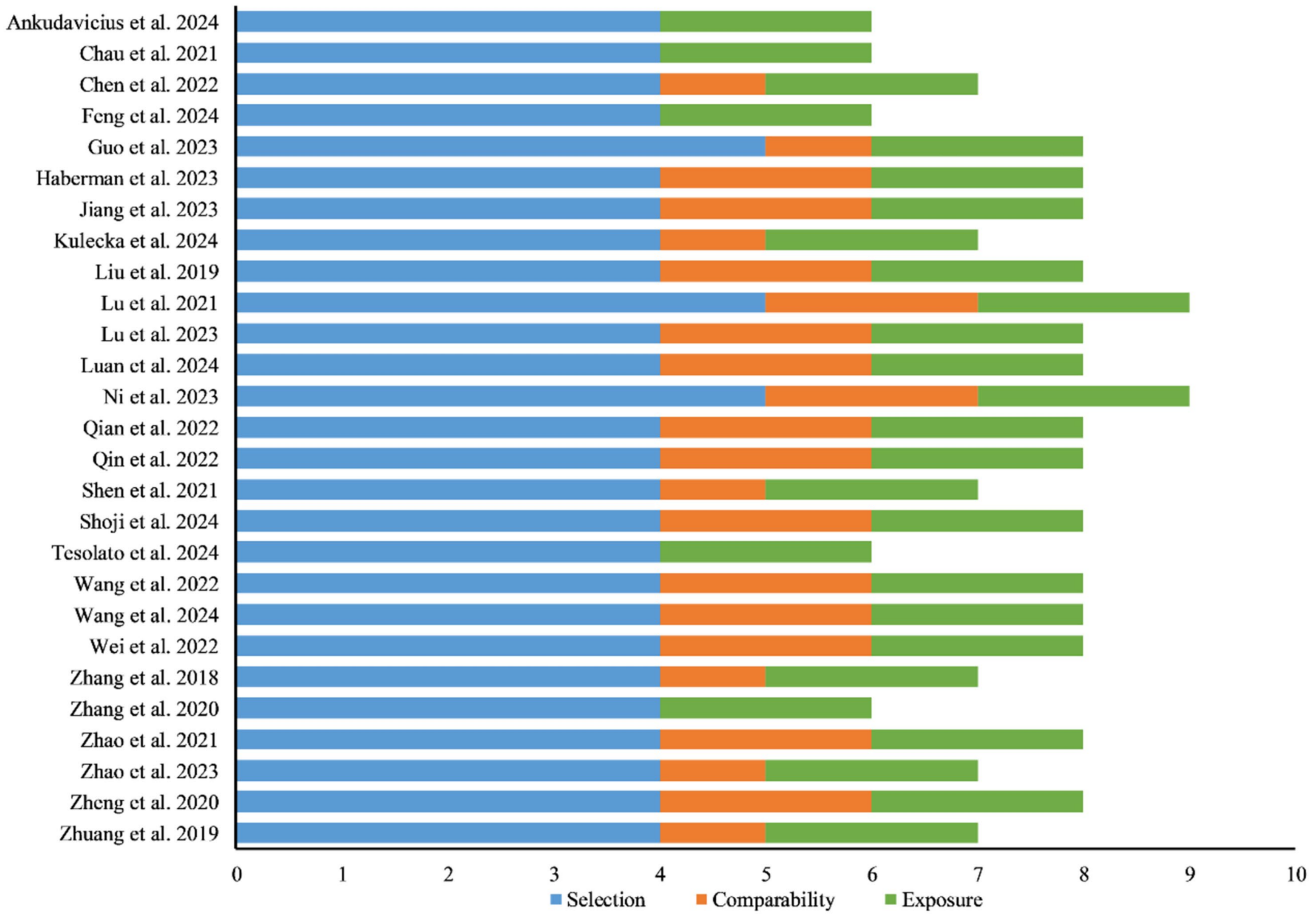
Quality score of included articles calculated using the NOS.

Methodological evaluation of the included studies was performed using the Newcastle-Ottawa Scale (NOS) (Stang, 2010). The quality assessment was independently conducted by two researchers using NOS, which assigns scores based on three main domains: (1) selection, (2) comparability, and (3) exposure. Any divergences between the two investigators were resolved through discussion with a third investigators. Studies were scored out of a maximum of 10 points, with the selection section contributing up to 5 points, comparability up to 2 points, and the results section up to 3 points. Studies achieving scores ranging from 7 to 10 were designated as high-quality research, whereas those scoring between 4 and 6 were categorized as moderate-quality research. Research endeavors scoring below 4 were deemed to be of low-quality. The scores assigned to each article by the NOS are presented in Figure 1; Supplementary Table 2.

### 2.6 Statistical analysis

Statistical analysis is performed on the relevant data of outcome indicators using Revman 5.3 software. For continuous variables, the mean difference (MD) or standardized mean difference (SMD) was utilized as the primary statistic for effect analysis, with the corresponding 95% confidence interval (CI) provided to quantify the precision of the estimate. If data were presented as the median accompanied by an interquartile range, we employed the recommended formula to transform those values into means with their corresponding standard deviations (Xiang Wan, 2014). If essential numerical data were unavailable, unclear, or could not be reliably extracted from the text, tables, figures, or Supplementary materials, the study was excluded from the corresponding quantitative synthesis and retained for descriptive analysis where appropriate. For qualitative data such as *β* diversity and relative abundance of microbial communities, the results of multiple studies were summarized in a table and a descriptive analysis was conducted. Statistical heterogeneity was assessed using the *I^2^* and *Tau^2^* statistic. An *I^2^* > 50% and either the *Tau^2^* > 0 was considered to indicate substantial heterogeneity, and therefore a random-effects model was applied to account for potential between-study variability in populations, sequencing methods, and microbiota assessment. After determining the appropriate model, the effect sizes included in the study were pooled, and forest plots were subsequently constructed to visually represent the data. The funnel plots served as a tool to detect publication bias. A well-symmetrical funnel plot indicated minimal publication bias, whereas asymmetry hinted at potential publication bias. Consequently, further quantitative assessment of the results was required, which was conducted using the Egger test implemented in Stata 17.0 software. We conducted sensitivity analysis through a one-by-one exclusion method to confirm the stability and reliability of our results. *p* < 0.05 was considered statistically significant.

## 3 Results

### 3.1 Search results and studies characteristics

**Figure 2:**
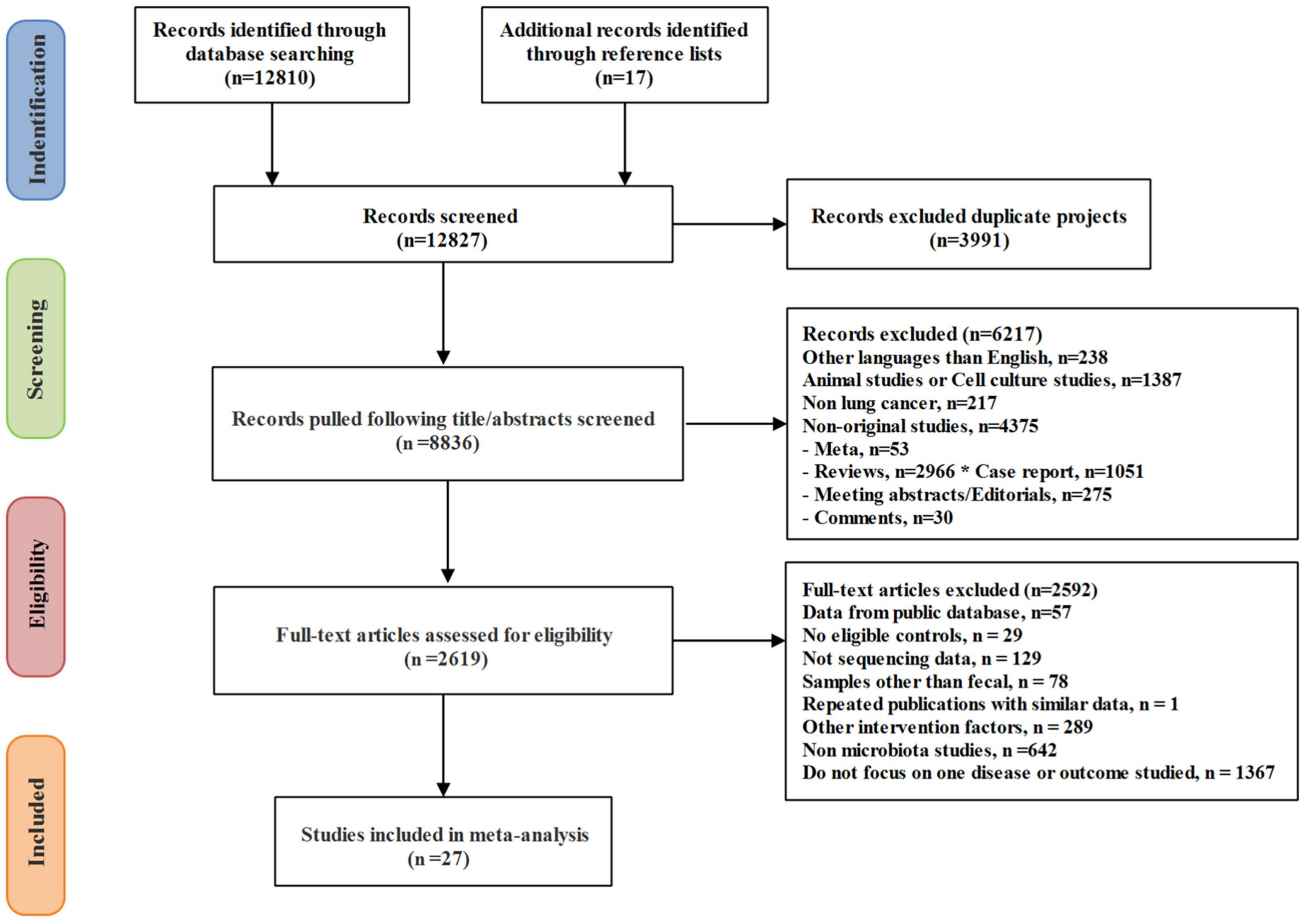
PRISMA-based study selection flow chart.

We obtained a total of 12,810 articles from four databases (PubMed, *n* = 2,196; Embase, *n* = 5,810; the Cochrane Library, *n* = 737; and Web of Science, *n* = 4,067). Additional records identified through reference lists (*n* = 17) were also included in our analysis. All records were imported into EndNote and, using EndNote software and manual filtering, duplicate studies (*n* = 3,991) were removed. Following the deduplication process, we further narrowed down our selection by screening the titles and abstracts of the remaining 8,836 articles. During this screening stage, 6,217 articles were excluded primarily due to the following reasons: they were published in languages other than English (*n* = 238), they did not focus on human studies (*n* = 1,387), they were not related to lung cancer (*n* = 217), or they were non-original studies (*n* = 4,375). Of the 2,619 full-text articles reviewed for eligibility, 2,592 were excluded primarily for the following reasons: their data were sourced from public databases (*n* = 57), they lacked eligible controls (*n* = 29), they were not sequencing data (*n* = 129), their samples were other than fecal specimens (*n* = 78), they were repeated publications with similar data (*n* = 1), they involved other intervention factors (*n* = 289), they were classified as non-microbiota studies (*n* = 642), and they did not focus on a single disease or outcome (*n* = 1,367). Ultimately, 27 studies met our inclusion criteria for systematic review. Figure 2 summarized a flow chart of the study selection procedure in its entirety.

The characteristics of the studies included in the meta-analysis were outlined in Table 1. This meta-analysis collected 2,263 individuals (1,234 LC patients and 1,029 healthy individuals/patients with benign pulmonary nodules) involving a total of 27 comparisons. The included studies were published between 2018 and 2025. Only one study enrolled a mixture of North American populations (Chau et al., 2021), while the remaining studies involved Asian populations. Among the included studies, lung cancer was judged based on international guidelines, taking into account pathological or cytological diagnosis. Among the included study population, 3 studies were from the community and 24 studies were from hospitals. Additionally, there was heterogeneity in the bacterial sequencing platforms used in these studies; specifically, 25 studies utilized Illumina© systems for sequencing: 12 used the Mi-Seq platform, 8 used the Nova-Seq platform, and 5 used the Hi-seq platform. One study did not specify the sequencing method. Moreover, 18 studies focused on the V3-V4 regions, identified as the most prevalent targets for amplifying 16S rRNA gene sequences.

**Table 1:** Basic characteristics of included studies.

Study	Year	Country/region	Setting	LC(n)	Non-LC controls		Age (case/control)	Gender (case/control)	16 s region	Sequencing platform	#NOS quality score (Max. of 10)
					BPN (n)	HC (n)					
Ankudavicius et al. (2024)	2024	Lithuania	Hospital	105 NSCLC		54	NM	NM	V1-V2	Illumina MiSeq platform	6
Chau et al. (2021)	2021	America	Hospital	34		32	68 (42–86)/ NM	Case: (M18/F16) Control: NM	V3-V4	Illumina MiSeq	6
Chen S. et al. (2022b)	2022	China	Hospital	30		15	58.73 ± 8.44/59.6 ± 5.45	Case: (M18/F12) Control: (M7/F8)	V3-V4	NovaSeq PE250	7
Feng et al. (2024)	2024	China	Hospital	12 NSCLC		9	56–70 63–83	NM	NM	Illumina HiSeq 2,500	6
Guo et al. (2023)	2023	China	Community	43 LADC		64	57 ± 11/46 ± 12	Case: (M21/F22) Control: (M36/F28)	V3-V4	Illumina NovaSeq 6,000	8
Haberman et al. (2023)	2023	Israel	Hospital	75		31	67(42–87)/ 67 (50–81)	Case: (M41/F34) Control: (M15/F16)	V4	Illumina MiSeq	8
Jiang et al. (2023)	2023	China	Hospital	80 NSCLC		35	56.95 ± 10.73/54.46 ± 8.98	Case: (M27/F53) Control: (M22/F13)	V4	Illumina HiSeq4000	8
Kulecka et al. (2024)	2024	Poland	Hospital	34		34	F 64(54-81)M 61(35–85)/ F 64(52-82)M 61(40–81)	Case: (M17/F17) Control: (M17/F17)	NM	Illumina NovaSeq 6,000	7
Liu et al. (2019)	2019	China	Hospital	30		16	59.68 ± 10.49/59.12 ± 7.76	Case: (M21/F9) Control: (M9/F7)	V4	Illumina HiSeq/MiniSeq	8
Lu et al. (2021)	2021	China	Community	85 NSCLC		29	58.65 ± 7.73/55.83 ± 12.04	Case: (M56/F29) Control: (M15/F14)	V3-V4	Illumina MiSeq	9
Lu et al. (2023)	2023	China	Hospital	52		29	56.92 ± 12.37/50.72 ± 16.54	Case: (M36/F16) Control: (M16/F13)	V3-V4	Illumina NovaSeq 6,000	8
Luan et al. (2024)	2024	China	Hospital	55	28		56.80 ± 17.39/57.71 ± 13.24	Case: (M28/F27) Control: (M17/F11)	V3-V4	Illumina MiSeq	8
Ni et al. (2023)	2023	China	Community	43 NSCLC		35	58.63 ± 9.92/55.8 ± 8.44	Case: (M18/F25) Control: (M18/F17)	V3-V4	NovaSeq PE250	9
Qian et al. (2022)	2022	China	Hospital	46 NSCLC		12	59.13 ± 9.42/60.75 ± 7.36	Case: (M21/F25) Control: (M6/F6)	NM	Illumina MiSeq	8
Qin et al. (2022)	2022	China	Hospital	61		28	54.54 ± 10.56/58.79 ± 11.16	Case: (M19/F42) Control: (M11/F17)	V3-V4	Illumina HiSeq/MiniSeq	8
Shen et al. (2021)	2021	China	Hospital	16		17	64 ± 7.4/64 ± 4.2	Case: (M13/F3) Control: (M15/F2)	V3-V4	NM	7
Shoji et al. (2024)	2024	Japan	Hospital	53		10	72 (50–89)/ 41(33–51)	Case: (M28/F25) Control: (M10/F0)	V3-V4	llumina MiSeq	8
Tesolato et al. (2024)	2024	Spain	Hospital	19 NSCLC		20	72.79 ± 7.91/54.80 ± 14.97	Case: (M9/F10) Control: (M6/F14)	V2, V4, V8, and V3, V6-7, V9,	Ion Torrent™	6
Wang et al. (2022)	2022	China	Hospital	45 LUAD		30	59.7 ± 10.20/58.5 ± 7.02	Case: (M24/F21) Control: (M16/F14)	V3-V4	Illumina NovaSeq	8
Wang et al. (2024)	2024	China	Hospital	49		18	57.73 ± 9.83/61.00 ± 7.05	Case: (M28/F21) Control: (M6/F12)	V3-V4	Illumina MiSeq PE300	8
Wei et al. (2022)	2022	Taiwan	Hospital	34 NSCLC		268	64.5 ± 8.9/64.1 ± 5.9	Case: (M20/F14) Control: (M113/F155)	V3-V4	Illumina Miseq	8
Zhang et al. (2018)	2018	China	Hospital	41		41	57.97 ± 7.68/59.05 ± 6.78	Case: (M30/F11) Control: (M26/F15)	V1-V2	Illumina MiSeq	7
Zhang et al. (2020)	2020	China	Hospital	60		17	63.4(47–77)/ NM	Case: (M41/F19) Control: NM	NM	Illumina HiSeq	6
Zhao et al. (2021)	2021	China	Hospital	39		40	<60 16/28 ≥60 23/12	Case: (M21/F18) Control: (M18/F22)	V3-V4	NovaSeq PE250	8
Zhao et al. (2023)	2023	China	Hospital	21 NSCLC		22	61.3 ± 9.6/33.2 ± 5.7	Case: (M18/F3) Control: (M8/F14)	V3-V4	Illumina NovaSeq	7
Zheng et al. (2020)	2020	China	Hospital	42		65	57.48 ± 12.49/58.60 ± 4.86	Case: (M18/F24) Control: (M10/F55)	V3-V4	Illumina MiSeq	8
Zhuang et al. (2019)	2019	China	Hospital	30		30	61 (52–72)/ 50 (19–95)	Case: (M12/F18) Control: (M10/F20)	V3-V4	Illumina MiSeq	7

HC, health control; BPN, benign pulmonary nodules; NM, not mentioned; **#**NOS, the Newcastle-Ottawa Scale.

When describing the microbial characteristics of the host intestine, researchers primarily utilized alpha diversity indices. Table 2 summarizes the findings of alpha diversity indices. 24 studies(Zhang et al., 2018; Liu et al., 2019; Zheng et al., 2020; Chau et al., 2021; Lu et al., 2021; Shen et al., 2021; Chen S. et al., 2022; Qian et al., 2022; Qin et al., 2022; Wang et al., 2022; Wei et al., 2022; Guo et al., 2023; Haberman et al., 2023; Jiang et al., 2023; Lu et al., 2023; Ni et al., 2023; Zhao et al., 2023; Ankudavicius et al., 2024; Feng et al., 2024; Kulecka et al., 2024; Luan et al., 2024; Shoji et al., 2024; Tesolato et al., 2024; Wang et al., 2024) reported at least one of the alpha diversity indexes, with the Shannon index being the most frequently reported (*n* = 21), followed closely by Chao1 richness (*n* = 17) and Simpson index (*n* = 16). Additionally, 27 studies (Zhang et al., 2018; Liu et al., 2019; Zhuang et al., 2019; Zhang et al., 2020; Zheng et al., 2020; Chau et al., 2021; Lu et al., 2021; Shen et al., 2021; Zhao et al., 2021; Chen S. et al., 2022; Qian et al., 2022; Qin et al., 2022; Wang et al., 2022; Wei et al., 2022; Guo et al., 2023; Haberman et al., 2023; Jiang et al., 2023; Lu et al., 2023; Ni et al., 2023; Zhao et al., 2023; Ankudavicius et al., 2024; Feng et al., 2024; Kulecka et al., 2024; Luan et al., 2024; Shoji et al., 2024; Tesolato et al., 2024; Wang et al., 2024) presented data on at least one beta diversity index. Furthermore, all studies (*n* = 27) provided information on the relative abundance of bacteria at the phylum or genus taxonomic levels.

**Table 2:** GRADE evidence profile.

Outcomes	No. of studies	Risk of bias	Inconsistency	Indirectness	Imprecision	Other considerations	Total number (intervention/control)	Certainty	Importance	SMD (95% CI)
ACE	7	Not serious	Serious^a^	Not serious	Not serious	None	688 (270/418)	⨁⨁⨁◯ Moderate^a^	Critical	−0.64 (−1.14,-0.13)
CHAO 1	17	Not serious	Serious^a^	Not serious	Not serious	None	1,399 (710/689)	⨁⨁⨁◯ Moderate^a^	Critical	−0.31 (−0.60,-0.02)
Observed species	12	Not serious	Serious^a^	Not serious	Serious^b^	None	959 (562/397)	⨁⨁◯◯ Low^a,b^	Important	−0.25 (−0.57, 0.08)
Pielou_evenness	5	Not Serious	Not serious	Not serious	Serious^b^	None	462 (273/189)	⨁⨁⨁◯ Moderate^b^	Critical	−0.10 (−0.51, 0.30)
Shannon	21	Not serious	Not serious	Not serious	Not serious	None	1786 (948/838)	⨁⨁⨁⨁ High	Critical	−0.26 (−0.44, −0.07)
Simpson	16	Not serious	Serious^a^	Not serious	Serious^b^	None	1,121 (743/378)	⨁⨁◯◯ Low^a,b^	Important	−0.14 (−0.41, 0.14)

CI, confidence interval; SMD, standardized mean difference.

^a^There was significant heterogeneity for ACE (I^2^ = 85%), CHAO 1 (I^2^ = 81%), observed species (I^2^ = 81%), and Simpson (I^2^ = 76%).

^b^The 95% CI crosses the equivalence line.

### 3.2 Quality of included studies

A total of 27 studies, including 1,234 LC patients and 1,029 non-lung cancer controls (1,001 HC and 28 BPN), were included in this meta-analysis. The quality of the 27 included studies was evaluated using the Newcastle-Ottawa Scale (NOS). Following the evaluation, 22 studies were rated as high-quality, 5 as medium-quality, and none were deemed poor-quality. The specific scores for each study based on the NOS are presented in Figure 1.

### 3.3 Alpha diversity indexes

**Figure 3:**
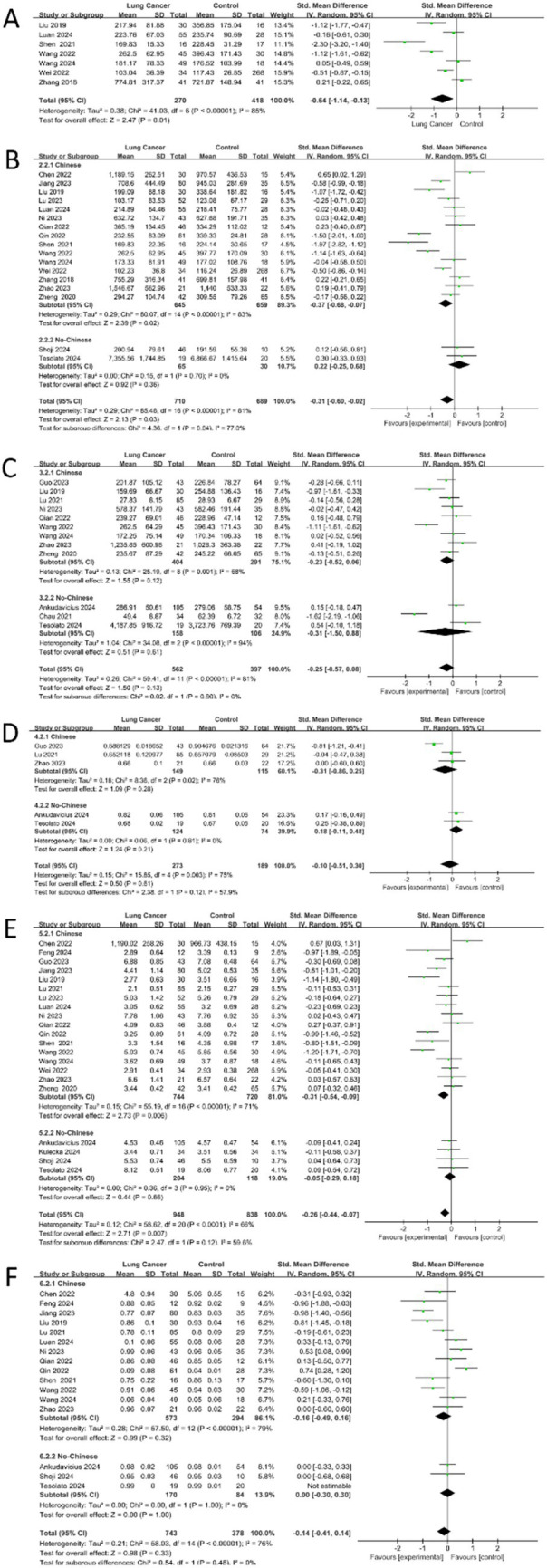
Forest plot comparing the alpha diversity between lung cancer and non-lung cancer groups in the included observational studies. **(A)** ACE index; **(B)** Chao 1 index; **(C)** Observed species index; **(D)** Pielou_evenness index; **(E)** Shannon index; **(F)** Simpson index. Differences between groups are presented as weights (percentages) and SMD (95% Cl). CI, confidence interval; IV, inverse variance; SD, standard deviation; SMD, standardized mean difference (Hedges’s g).

Alpha diversity serves as a predictive indicator of both species richness and evenness, reflecting both the number of species and their individual distribution. In all the included articles, 25 studies reported on alpha diversity. We employed the ACE index, Chao1 index, and observed species index to assess microbial richness, whereas the Shannon index and Simpson index were utilized to evaluate microbial evenness. Among the studies included in our analysis, 7 studies (Zhang et al., 2018; Liu et al., 2019; Shen et al., 2021; Wang et al., 2022; Wei et al., 2022; Luan et al., 2024; Wang et al., 2024) compared the ACE index between lung cancer (LC) and non-LC controls. 17 studies (Zhang et al., 2018; Liu et al., 2019; Zheng et al., 2020; Shen et al., 2021; Chen S. et al., 2022; Qian et al., 2022; Qin et al., 2022; Wang et al., 2022; Wei et al., 2022; Jiang et al., 2023; Lu et al., 2023; Ni et al., 2023; Zhao et al., 2023; Luan et al., 2024; Shoji et al., 2024; Tesolato et al., 2024; Wang et al., 2024) reported on the Chao1 index for quality assessment purposes. Similarly, 10 studies (Liu et al., 2019; Zheng et al., 2020; Chau et al., 2021; Qian et al., 2022; Wang et al., 2022; Guo et al., 2023; Ni et al., 2023; Zhao et al., 2023; Tesolato et al., 2024; Wang et al., 2024) provided data on the Observed species index and 5 studies (Lu et al., 2021; Guo et al., 2023; Zhao et al., 2023; Ankudavicius et al., 2024; Tesolato et al., 2024) provided data on the Pielou_evenness index. A total of 21 studies(Liu et al., 2019; Zheng et al., 2020; Lu et al., 2021; Shen et al., 2021; Qian et al., 2022; Qin et al., 2022; Wang et al., 2022; Wei et al., 2022; Guo et al., 2023; Jiang et al., 2023; Lu et al., 2023; Ni et al., 2023; Zhao et al., 2023; Ankudavicius et al., 2024; Feng et al., 2024; Kulecka et al., 2024; Luan et al., 2024; Shoji et al., 2024; Tesolato et al., 2024; Wang et al., 2024) reported the Shannon index, while 16 studies (Liu et al., 2019; Lu et al., 2021; Shen et al., 2021; Chen S. et al., 2022; Qian et al., 2022; Qin et al., 2022; Wang et al., 2022; Jiang et al., 2023; Ni et al., 2023; Zhao et al., 2023; Ankudavicius et al., 2024; Feng et al., 2024; Luan et al., 2024; Shoji et al., 2024; Tesolato et al., 2024; Wang et al., 2024) presented data on the Simpson index. We conducted random-effects meta-analyses, with the results illustrated in Figure 3.

Based on quantitative meta-analysis by the forest plot, it was evident that indicated lower alpha diversity indices in the observation group compared to the control group. Our analysis revealed substantial heterogeneity for the ACE index (*Tau^2^* = 0.38, *I^2^* = 85%, *p* < 0.00001), with a significant lower in lung cancer patients than those of non-lung cancer individuals (SMD = −0.64, 95% CI: −1.14 to −0.13, *p* = 0.01; Figure 3A). Consistently, the CHAO 1 index showed significant heterogeneity (*Tau^2^* = 0.29, *I^2^* = 81%, *p* < 0.00001) and a significant lower in lung cancer group (SMD = −0.31, 95% CI: −0.60 to −0.02, *p* = 0.03; Figure 3B). In contrast, the Observed species index exhibited substantial heterogeneity (*Tau^2^* = 0.26, *I^2^* = 81%, *p* < 0.00001) but were not significantly different between groups (SMD = −0.25, 95% CI: −0.57 to 0.08, *p* = 0.13; Figure 3C), although lung cancer patients tended to have lower species counts. Similarly, the Pielou evenness index measured the degree of uniformity in the distribution of species (*Tau*^2^ = 0.15, *I*^2^ = 75%, *p* = 0.003) and showed no significant difference (SMD = −0.10, 95% CI: −0.51 to 0.30, *p* = 0.61; Figure 3D). The lung cancer group also exhibited a lower Shannon index compared to the control group (SMD = −0.26, 95% CI: −0.44 to −0.07, *p* = 0.007; Figure 3E), with a significant heterogeneity (*Tau*^2^ = 0.12, *I*^2^ = 66%, *p* < 0.0001). However, no significant difference in the Simpson index was observed between the lung cancer and non-lung cancer patients (SMD = −0.14, 95% CI: −0.41 to 0.14, *p* = 0.33; Figure 3F). Furthermore, subgroup analysis based on population origin indicated that studies conducted in Chinese cohorts showed significantly lower Chao1 (SMD = −0.37, 95% CI: −0.68 to −0.07, *p* = 0.02; Figure 3B) and Shannon (SMD = −0.31, 95% CI: −0.54 to −0.09, *p* = 0.006; Figure 3E) indices in lung cancer group, suggesting a reduction in gut microbiota alpha diversity in lung cancer patients.

### 3.4 Beta diversity indexes

The *β* diversity index measures species diversity between ecosystems by comparing taxonomic units. It is used to quantify the differences between communities. In all the included articles, 27 studies reported on beta diversity. Some studies employed multiple methods for beta diversity analysis. Among all the included articles, 21 studies (Zhang et al., 2018; Liu et al., 2019; Zhuang et al., 2019; Zheng et al., 2020; Chau et al., 2021; Shen et al., 2021; Zhao et al., 2021; Chen S. et al., 2022; Qin et al., 2022; Wang et al., 2022; Wei et al., 2022; Guo et al., 2023; Haberman et al., 2023; Jiang et al., 2023; Lu et al., 2023; Ni et al., 2023; Zhao et al., 2023; Feng et al., 2024; Kulecka et al., 2024; Luan et al., 2024; Shoji et al., 2024) showed significant differences in gut microbiota beta diversity between lung cancer (LC) and non-lung cancer groups. Among them, 18 articles (Zhang et al., 2018; Liu et al., 2019; Zheng et al., 2020; Shen et al., 2021; Zhao et al., 2021; Qin et al., 2022; Wang et al., 2022; Wei et al., 2022; Guo et al., 2023; Haberman et al., 2023; Jiang et al., 2023; Lu et al., 2023; Ni et al., 2023; Zhao et al., 2023; Feng et al., 2024; Shoji et al., 2024) used Principal Coordinate Analysis (PCoA), 5 articles (Liu et al., 2019; Chen S. et al., 2022; Qin et al., 2022; Wang et al., 2022; Kulecka et al., 2024) used Principal Component Analysis (PCA), 4 articles (Liu et al., 2019; Zhuang et al., 2019; Jiang et al., 2023; Luan et al., 2024) used NMDS, and 2 articles (Chau et al., 2021; Luan et al., 2024) used PLS-DA (Table 3). The most commonly used methods were PCoA analysis based on unweighted UniFrac distance and Bray–Curtis dissimilarity. The principal coordinate analyses based on unweighted UniFrac distance were most frequently conducted (*n* = 11), of which one study revealed no significant differences (Qian et al., 2022), while 10 studies revealed significant differences between LC and non-LC groups (Zhang et al., 2018; Zheng et al., 2020; Zhao et al., 2021; Qin et al., 2022; Wei et al., 2022; Guo et al., 2023; Haberman et al., 2023; Jiang et al., 2023; Zhao et al., 2023; Shoji et al., 2024). The principal coordinate analyses based on Bray–Curtis dissimilarity were also frequently conducted (*n* = 9), of which one study revealed no significant differences (Lu et al., 2021), while seven studies revealed significant differences between LC and non-LC groups (Zheng et al., 2020; Shen et al., 2021; Zhao et al., 2021; Wang et al., 2022; Guo et al., 2023; Lu et al., 2023; Ni et al., 2023; Shoji et al., 2024).

**Table 3:** Summary of beta diversity assessments in the included studies.

Study	β diversity	Findings	Statistic value
Ankudavicius et al. (2024)	PCoA based on Bray-Curtis distance matrix	No significant difference in gut microbial composition among LC and HC	NM
Chau et al. (2021)	PLS-DA	A significant difference in gut microbial composition among LC and HC	NM
Chen S. et al. (2022b)	PCA based on the weighted UniFrac distance	A significant difference in gut microbial composition among LC and HC	*p* = 0.021
Feng et al. (2024)	PCoA based on Bray-Curtis distance	A significant difference in gut microbial composition among LC and HC	*p* = 0.0076
Guo et al. (2023)	PCoA based on unweighted UniFrac distances	A significant difference in gut microbial composition among LC and HC	*p* = 0.001
	PCoA based on Bray–Curtis dissimilarity	A significant difference in gut microbial composition among LC and HC	*p* = 0.002
Haberman et al. (2023)	PCoA based on unweighted UniFrac distance	A significant difference in gut microbial composition among LC and HC	*P* = 0.001
Jiang et al. (2023)	PCoA based on unweighted UniFrac distance	A significant difference in gut microbial composition among LC and HC	*p* = 0.025
	NMDS based on Bray-Curtis distances	A significant difference in gut microbial composition among LC and HC	stress = 0.107
Kulecka et al. (2024)	PCA	A significant difference in gut microbial composition among LC and HC	*p* = 0.001
Liu et al. (2019)	PCA based on unweighted UniFrac distance	A significant difference in gut microbial composition among LC and HC	*P* = 0.001
	PCoA based on weighted UniFrac distance	A significant difference in gut microbial composition among LC and HC	NM
	NMDS based on Bray-Curtis distances	A significant difference in gut microbial composition among LC and HC	NM
Lu et al. (2021)	PCoA based on Bray–Curtis dissimilarity	No significant difference in gut microbial composition among LC and HC	*p* = 0.079
Lu et al. (2023)	PCoA based on Bray–Curtis dissimilarity	A significant difference in gut microbial composition among LC and HC	*p* = 0.017
Luan et al. (2024)	NMDS	No significant difference in gut microbial composition among LC and BPN	stress = 0.254
	PLS-DA	A significant difference in gut microbial composition among LC and BPN	NM
Ni et al. (2023)	PCoA based on Bray–Curtis dissimilarity	A significant difference in gut microbial composition among LC and HC	*p* = 0.034
Qian et al. (2022)	PCoA based on unweighted UniFrac distance	No significant difference in gut microbial composition among LC and HC	NM
	PCoA based on weighted UniFrac distances	No significant difference in gut microbial composition among LC and HC	NM
Qin et al. (2022)	PCA based on unweighted UniFrac distance	A significant difference in gut microbial composition among LC and HC	*p* = 0.007
	PCoA based on unweighted UniFrac distance	A significant difference in gut microbial composition among LC and HC	*p* = 0.001
Shen et al. (2021)	PCoA based on Bray–Curtis dissimilarity	A significant difference in gut microbial composition among LC and HC	*p* = 0.001
Shoji et al. (2024)	PCoA based on Bray–Curtis dissimilarity	A significant difference in gut microbial composition among LC and HC	*p* = 0.005
	PCoA based on unweighted UniFrac distances	A significant difference in gut microbial composition among LC and HC	*p* = 0.002
	PCoA based on weighted UniFrac distances	A significant difference in gut microbial composition among LC and HC	*p* = 0.002
Tesolato et al. (2024)	PCoA analysis based on Jaccard distance algorithm	No significant difference in gut microbial composition among LC and HC	*p* = 0.082
Wang et al. (2022)	PCoA based on Bray–Curtis dissimilarity	A significant difference in gut microbial composition among LC and HC	NM
	PCA	A significant difference in gut microbial composition among LC and HC	NM
Wang et al. (2024)	PCoA based on the Bray_Curtis distance	No significant difference in gut microbial composition among LC and HC	*p* = 0.709
Wei et al. (2022)	PCoA based on unweighted UniFrac distance	A significant difference in gut microbial composition among LC and HC	*p* < 0.001
	PCoA based on weighted UniFrac distances	A significant difference in gut microbial composition among LC and HC	*p* < 0.001
Zhang et al. (2018)	PCoA based on unweighted UniFrac distances	A significant difference in gut microbial composition among LC and HC	*p* = 0.001
	PCoA based on the weighted UniFrac distance	No significant difference in gut microbial composition among LC and HC	NM
Zhang et al. (2020)	NM	NM	NM
Zhao et al. (2021)	PCoA based on unweighted UniFrac distance	A significant difference in gut microbial composition among LC and HC	*p* = 0.001
	PCoA based on Bray–Curtis dissimilarity	A significant difference in gut microbial composition among LC and HC	*p* = 0.006
Zhao et al. (2023)	PCoA based on unweighted UniFrac distance	A significant difference in gut microbial composition among LC and HC	*p* = 0.002
	PCoA based on Jaccard distance	A significant difference in gut microbial composition among LC and HC	*p* = 0.001
Zheng et al. (2020)	PCoA based on unweighted UniFrac distance	A significant difference in gut microbial composition among LC and HC	*p* = 0.003
	PCoA based on Bray–Curtis dissimilarity	A significant difference in gut microbial composition among LC and HC	*p* = 0.007
Zhuang et al. (2019)	NMDS based on weighted UniFrac distance	A significant difference in gut microbial composition among LC and HC	stress = 0.153

NM, not mentioned.

### 3.5 Relative abundance of microbial taxa

**Figure 4:**
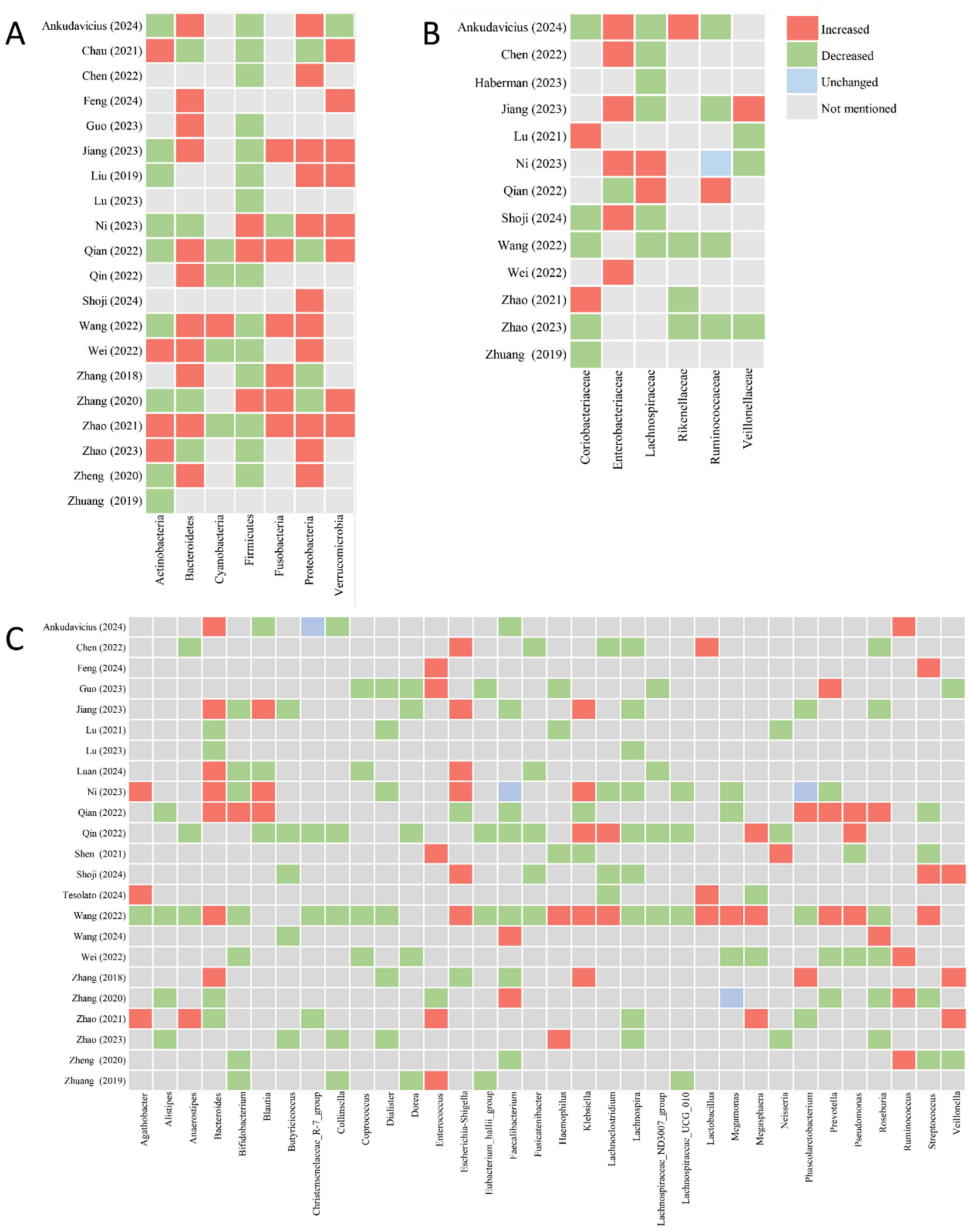
Taxa relative abundance changes in gut microbiota between lung cancer patients compared to non-lung cancer controls. **(A)** Heatmap analysis at the phylum level; **(B)** Heatmap analysis at the family level; **(C)** Heatmap analysis at the genus level.

**Figure 5:**
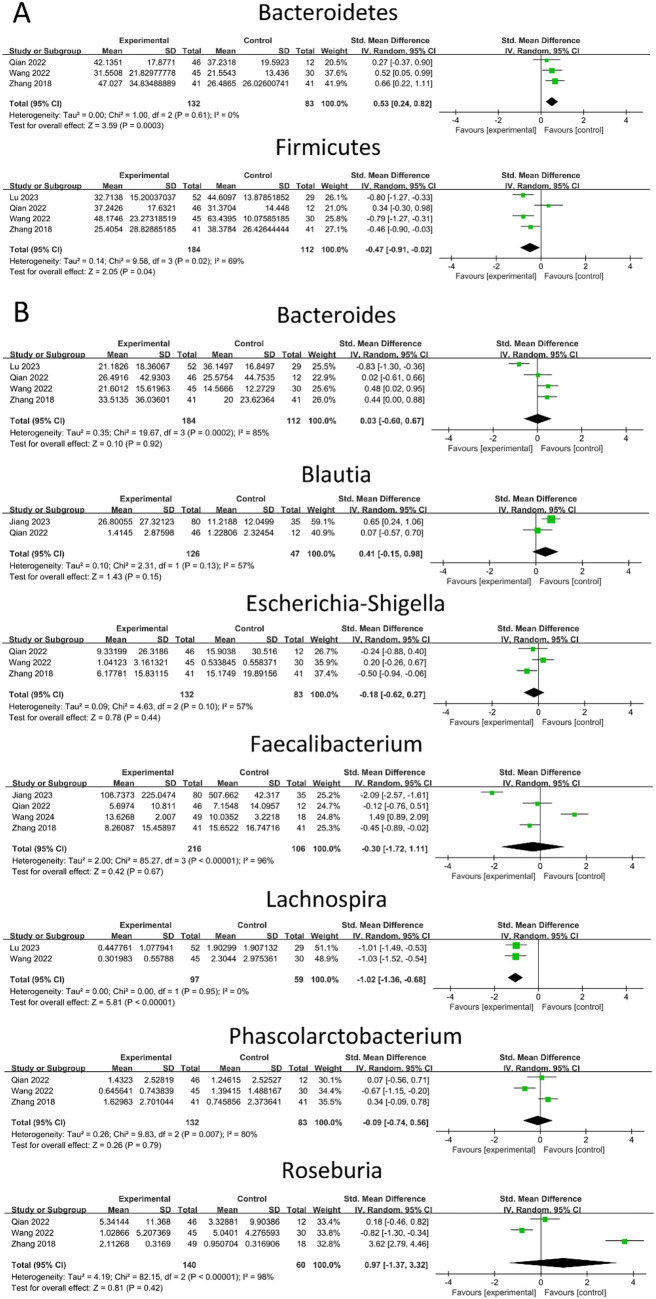
Forest plot comparing abundance in 2 phyla and 7 genera of gut microbiota between lung cancer patients and non-lung cancer controls in the included observational studies. **(A)** 2 phyla: Bacteroidetes and Firmicutes; **(B)** 7 genera: *Bacteroides*, *Blautia*, *Escherichia-Shigella*, *Faecalibacterium*, *Lachnospira*, *Phascolarctobacterium*, *Roseburia*. Differences between groups are presented as weights (percentages) and SMD (95% Cl). CI, confidence interval; IV, inverse variance; SD, standard deviation; SMD, standardized mean difference (Hedges’s g).

Most studies have illustrated the comparative abundance of microbial phyla, families, and genera in both LC and non-LC groups through graphical displays. We observed significant alterations in the microbial communities as portrayed in the images, and subsequently compiled the essential insights derived from these studies (depicted in Figures 4, 5). 20 articles (Zhang et al., 2018; Liu et al., 2019; Zhuang et al., 2019; Zhang et al., 2020; Zheng et al., 2020; Chau et al., 2021; Zhao et al., 2021; Chen S. et al., 2022; Qian et al., 2022; Qin et al., 2022; Wang et al., 2022; Wei et al., 2022; Guo et al., 2023; Jiang et al., 2023; Lu et al., 2023; Ni et al., 2023; Zhao et al., 2023; Ankudavicius et al., 2024; Feng et al., 2024; Shoji et al., 2024) analyzed the taxa at the phylum level (Figure 4A). Upon synthesizing these research findings at the phylum level, we observed significant differences in the gut microbiota between the lung cancer group and the non-lung cancer group. Specifically, compared with the non-lung cancer group, the lung cancer group exhibited decreased relative abundances of Firmicutes (82%, 14/17), Cyanobacteria (80%, 4/5), and Actinobacteria (69%, 9/13). Conversely, the relative abundances of Verrucomicrobia (88%, 8/9), Fusobacteria (86%, 6/7), Bacteroidetes (73%, 11/15), and Proteobacteria (73%, 11/15), were increased in the lung cancer group. 4 studies (Zhang et al., 2018; Lu et al., 2023; Qian et al., 2022; Wang et al., 2022) provided sufficient quantitative data to calculate the relative abundances of Firmicutes. Quantitative meta-analysis by forest plot revealed substantial heterogeneity for the relative abundances of Firmicutes (*Tau^2^* = 0.14, *I^2^* = 69%, *p* = 0.02), with a significant lower in lung cancer patients than those of non-lung cancer individuals (SMD = −0.47, 95% CI: −0.91 to −0.02, *p* = 0.04; Figure 5A). 3 studies (Zhang et al., 2018; Qian et al., 2022; Wang et al., 2022) provided sufficient quantitative data to calculate the relative abundances of Bacteroidetes. In contrast, the relative abundances of Bacteroidetes (*Tau^2^* = 0.00, *I^2^* = 0%, *p* = 0.61) showed a significant increase in lung cancer group (SMD = 0.53, 95% CI: 0.24 to 0.82, *p* = 0.0003; Figure 5A).

13 articles (Zhuang et al., 2019; Lu et al., 2021; Zhao et al., 2021; Chen S. et al., 2022; Qian et al., 2022; Wang et al., 2022; Wei et al., 2022; Haberman et al., 2023; Jiang et al., 2023; Ni et al., 2023; Zhao et al., 2023; Ankudavicius et al., 2024; Shoji et al., 2024) analyzed the taxa at the family level (Figure 4B). Upon aggregate analysis, we observed that, compared with the non-lung cancer group, the lung cancer group exhibited decreased relative abundances of Lachnospiraceae (75%, 6/8), Rikenellaceae (75%, 3/4), Veillonellaceae (75%, 3/4), Coriobacteriaceae (71%, 5/7), and Ruminococcaceae (67%, 4/6). Conversely, the relative abundances of Enterobacteriaceae (86%, 6/7) was increased in the lung cancer group.

Furthermore, the most prevalent studies were those reporting results at the genus level. 23 articles (Zhang et al., 2018; Zhuang et al., 2019; Zhang et al., 2020; Zheng et al., 2020; Lu et al., 2021; Shen et al., 2021; Zhao et al., 2021; Chen S. et al., 2022; Qian et al., 2022; Qin et al., 2022; Wang et al., 2022; Wei et al., 2022; Guo et al., 2023; Jiang et al., 2023; Lu et al., 2023; Ni et al., 2023; Zhao et al., 2023; Ankudavicius et al., 2024; Feng et al., 2024; Luan et al., 2024; Shoji et al., 2024; Tesolato et al., 2024; Wang et al., 2024) analyzed the taxa at the genus level (Figure 4C). Our aggregate analysis revealed that, in comparison to the non-lung cancer group, the lung cancer group showed decreased relative abundances of *Lachnospira* (100%, 9/9), *Dialister* (100%, 6/6), *Dorea* (100%, 5/5), *Collinsella* (100%, 5/5), *Fusicatenibacter* (100%, 5/5), *Butyricicoccus* (100%, 5/5), *Coprococcus* (100%, 4/4), *Alistipes* (100%, 4/4), *Eubacterium_hallii_group* (100%, 4/4), *Lachnospiraceae_UCG_010* (100%, 4/4), *Lachnospiraceae_ND307_group* (100%, 4/4), *Bifidobacterium* (88%, 7/8), *Roseburia* (75%, 6/8), *Anaerostipes* (75%, 3/4), *Christensenelaceae_R-7_group* (75%, 3/4), *Neisseria* (75%, 3/4), *Faecalibacterium* (70%, 7/10), *Lachnoclostridium* (67%, 4/6), *Haemophilus* (60%, 3/5), *Megamonas* (60%, 3/5), and *Streptococcus* (57%, 4/7). In contrast, the lung cancer group exhibited increased relative abundances of *Ruminococcus* (100%, 4/4), *Lactobacillus* (100%, 3/3), *Enterococcus* (83%, 5/6), *Escherichia-Shigella* (75%, 6/8), *Agathobacter* (75%, 3/4), *Klebsiella* (71%, 5/7), *Bacteroides* (60%, 6/10), *Megasphaera* (60%, 3/5), *Pseudomonas* (60%, 3/5), *Veillonella* (60%, 3/5), and *Blautia* (60%, 3/5). 4 studies (Zhang et al., 2018; Lu et al., 2023; Qian et al., 2022; Wang et al., 2022) provided sufficient quantitative data to calculate the relative abundances of *Bacteroides*, 2 studies (Qian et al., 2022; Jiang et al., 2023) to calculate the relative abundances of *Blautia*, 3 studies (Zhang et al., 2018; Qian et al., 2022; Wang et al., 2022) to calculate the relative abundances of *Escherichia-Shigella*, 4 studies (Zhang et al., 2018; Jiang et al., 2023; Qian et al., 2022; Wang et al., 2022) to calculate the relative abundances of *Faecalibacterium*, 2 studies (Lu et al., 2023; Wang et al., 2022) to calculate the relative abundances of *Lachnospira*, 3 studies (Zhang et al., 2018; Qian et al., 2022; Wang et al., 2022) to calculate the relative abundances of *Phascolarctobacterium*, and 3 studies (Zhang et al., 2018; Qian et al., 2022; Wang et al., 2022) to calculate the relative abundances of *Roseburia*. Based on the quantitative meta-analysis, the relative abundances of *Lachnospira* (*Tau^2^* = 0.00, *I^2^* = 0%, *p* = 0.95) was significantly lower in lung cancer group (SMD = −1.01, 95% CI: −1.29 to −0.73, *p* < 0.00001; Figure 5B). However, no significant difference in the relative abundances of *Bacteroides*, *Blautia*, *Escherichia-Shigella*, *Faecalibacterium*, *Phascolarctobacterium*, *and Roseburia* were observed between the lung cancer and non-lung cancer patients.

Five studies identified the gut microbiota markers distinguishing lung cancer from healthy controls (Shen et al., 2021; Wang et al., 2022; Wei et al., 2022; Jiang et al., 2023; Tesolato et al., 2024). The gut microbiota markers include one bacterium and a model containing multiple bacteria. Two studies reported the consistent genera *Streptococcus* and *Klebsiella*, in which the AUCs were between 0.675–0.935 for *Streptococcus* and 0.755–0.957 for *Klebsiella,* with a sensitivity of 70.6–88.2% for *Streptococcus* and 88.2% for *Klebsiella*, and a specificity of 91.9% for *Streptococcus* and 94.6% for *Klebsiella* (Shen et al., 2021; Jiang et al., 2023). Genus *Bacteroides* and its species were shown in 2 studies, with the AUCs 0.767 for *Bacteroides and* 0.669 for *Bacteroides caccae*. Other gut microbiota markers were varied among different studies (Wang et al., 2022; Wei et al., 2022). The AUCs were between 0.639–0.959, with a sensitivity between 70.6–94.1%, and a specificity between 64.9–100% (Supplementary Table 3).

### 3.6 Risk assessment of bias in meta-analysis

The result for the heterogeneity assessment showed that *I^2^* was >50% and either the *Tau^2^* > 0, indicating strong heterogeneity. A sensitivity analysis was performed, sequentially excluding individual studies to observe changes in pooled results, validating the accuracy and stability of the findings (Supplementary Figure 1). Sensitivity analysis of ACE, Chao1, Shannon, and Simpson indices revealed no significant changes upon excluding individual studies, suggesting result stability. Sensitivity analysis of the Observed species index revealed significant differences between the lung cancer and control groups after excluding the studies by Qian et al. (2022) or Zhao et al. (2023). This difference may be attributed to the large disparity in sample size between the control group and the lung cancer group in these two studies. To validate our reliability hypothesis, we evaluated publication bias using funnel plot symmetry and Egger’s test. The symmetry of the funnel plot showed no evidence of publication bias (Supplementary Figure 2). Similarly, Egger’s test also indicated no significant publication bias for the ACE index (*p* = 0.14), Chao 1 index (*p* = 0.91), Observed species index (*p* = 0.54), Pielou’s evenness index (*p* = 0.85), Shannon index (*p* = 0.52), and Simpson index (*p* = 0.45) (Supplementary Table 4). These results suggest that the conclusions of the meta-analysis are relatively stable.

## 4 Discussion

Gut microbial dysbiosis may contribute to lung cancer development and progression through several interconnected pathways. Firstly, the reduction in SCFA-producing bacteria leads to a pro-inflammatory state and increased gut permeability, allowing microbial components such as lipopolysaccharide (LPS) to translocate into systemic circulation and stimulate Toll-like receptor (TLR)-mediated signaling in the lungs (Shen et al., 2023; Verma et al., 2024). This can result in chronic inflammation, a known driver of oncogenesis. Secondly, the enrichment of potentially pathogenic taxa like *Escherichia coli* and *Enterococcus faecalis* may promote the generation of reactive oxygen species and genotoxic metabolites, contributing to DNA damage and tumor-promoting microenvironments (Yang et al., 2023; Montanari et al., 2025). Moreover, alterations in bile acid metabolism and amino acid fermentation pathways observed in metabolomic studies suggest that microbial dysbiosis may influence host metabolic signaling in ways that favor tumor proliferation and immune evasion (Collins et al., 2023; Ni et al., 2023).

Our comprehensive meta-analysis systematically examined gut microbiota between LC patients and healthy individuals and patients with BPNs. We integrate data from 27 studies to investigate the composition alteration in microbiota diversity and microbiota abundance at the phylum, family and genus levels across the gut in a cohort of 2,263 individuals (1,234 LC patients and 1,029 healthy individuals/patients with benign pulmonary nodules). We assessed the gut microbiota using measures such as alpha diversity index, beta diversity, and microbial group abundance to evaluate alteration in microbiota composition. Our findings reveal consistent and significant alterations in microbial diversity and composition, suggesting a strong association between gut microbiota dysbiosis and lung cancer. This work highlights the potential of the gut microbiome as both a biomarker source and a biological contributor to lung carcinogenesis, progression, and treatment response.

Alpha diversity is a fundamental metric for evaluating microbial ecological balance, capturing the variety and distribution of species within a given microbial community at specific spatial or temporal points (Cassol et al., 2025). Commonly applied indices of alpha diversity include Chao1, ACE, Shannon, and Simpson, each highlighting different ecological attributes. Specifically, the Chao1 and ACE indices quantify species richness, offering insights into the number of distinct taxa present. In contrast, the Shannon and Simpson indices account for both richness and evenness, thereby reflecting the overall uniformity and distribution of microbial populations within the community (Kim et al., 2017). One of the most significant findings was a marked reduction in microbial *α*-diversity among lung cancer patients compared to healthy controls, particularly the ACE, Chao1, and Shannon indices. Alpha diversity reduction suggests a disrupted gut microbial ecosystem in lung cancer. This aligns with prior research demonstrating that microbial diversity is often compromised in the context of lung malignancy (Zhuang et al., 2019). Reduced microbial diversity can compromise colonization resistance, metabolic balance, and immune homeostasis, potentially creating a microenvironment conducive to cancer development and progression (Yang et al., 2023).

Consistently, our meta-analysis demonstrated significant differences in beta diversity of most studies, further reinforcing that the gut microbial composition in LC patients is distinct from that of non-cancer controls. However, the beta-diversity findings should be interpreted cautiously because the included studies used heterogeneous analytical methods and distance metrics. PCoA and NMDS were based on different ecological distance matrices, whereas PCA and PLS-DA assess data structure using different statistical assumptions and are not directly equivalent to distance-based beta-diversity analyses. Consequently, the beta-diversity evidence could not be synthesized as a unified quantitative outcome.

At the taxonomic level, our findings reveal a depletion of commensal, health-promoting bacterial taxa in LC patients. Genera such as *Faecalibacterium*, *Bifidobacterium*, *Roseburia*, and *Lachnospira* were consistently reduced, which all known for their anti-inflammatory and immunomodulatory roles. Conversely, an enrichment of potentially pathogenic or pro-inflammatory genera, including *Escherichia-Shigella*, *Enterococcus*, *Klebsiella*, and *Bacteroides*, was observed. However, only *Lachnospira* showed a statistically significant reduction in the quantitative meta-analysis. As a genus that includes short-chain fatty acid-producing bacteria, its lower reported relative abundance may suggest altered microbial metabolic capacity in patients with lung cancer (Jiang et al., 2023). Nevertheless, this finding should still be interpreted cautiously because the analysis was based on relative-abundance data from heterogeneous studies using different populations, sequencing platforms, amplified regions, and bioinformatic pipelines.

Recent genetic and clinical studies provide additional context for interpreting the relationship between the gut microbiota and lung cancer. Mendelian-randomization analyses have identified several genetically predicted microbial traits associated with the risk of lung cancer and its histological subtypes (Li et al., 2023; Chen J. et al., 2024; Chen Z. et al., 2024). Some studies have suggested that lower genetically predicted abundance of selected commensal taxa may be associated with higher NSCLC risk. These findings are broadly compatible with the hypothesis that disruption of immunoregulatory or metabolically beneficial microbial communities may contribute to lung carcinogenesis. The microbiome may also represent a therapeutic target rather than solely an observational biomarker. A recent study of patients with advanced NSCLC receiving chemoimmunotherapy reported that concomitant administration of the live bacterial product *Clostridium butyricum* MIYAIRI 588 (CBM588) was associated with longer overall survival (Tomita et al., 2023; Yang et al., 2025). This observation provides preliminary clinical support for microbiota-directed modulation during systemic therapy. Smoking represents another major limitation in interpreting the current evidence. Tobacco exposure is not only the dominant risk factor for lung cancer but is also associated with changes in oral, airway, and gut microbial composition (Fan et al., 2023; Miyauchi et al., 2025; Zhang et al., 2025). Recent population-based and Mendelian-randomization studies further suggest bidirectional relationships between smoking-related traits and selected gut microbial taxa (Fan et al., 2023; Miyauchi et al., 2025; Zhang et al., 2025). Because smoking status, pack-years, current versus former smoking, and time since cessation were inconsistently reported across the included studies, residual confounding could not be adequately addressed. Thus, some of the microbial differences observed between patients with lung cancer and controls may reflect tobacco exposure, smoking-related respiratory disease, dietary differences, or associated medications rather than lung cancer itself.

These findings have important clinical implications. First, gut microbiota profiles may serve as non-invasive biomarkers for early lung cancer detection or for distinguishing malignant from benign pulmonary nodules. Second, specific taxa could be leveraged to predict or even enhance therapeutic response, particularly to immunotherapies. Third, microbiota-targeted interventions, including prebiotics, probiotics, synbiotics, and fecal microbiota transplantation, could represent adjunctive strategies to improve lung cancer outcomes. However, these translational prospects require validation in large, controlled trials.

Several limitations should be considered when interpreting these findings. First, substantial heterogeneity existed across the included studies in terms of study design, participant source, and control selection, with most studies being cross-sectional and therefore unable to support causal inference. Second, the geographic coverage was limited, as 24 included studies were conducted in Asian populations, only 3 studies included the European population, and only 1 study included a mixed North American population, which may restrict the generalizability of the findings. Third, an apparent change in one taxon may result partly from changes in other taxa and does not necessarily indicate a change in its absolute abundance. Although SMD allowed us to combine published continuous outcomes reported on different numerical scales, it did not correct for compositionality. In addition, methodological heterogeneity in microbiome assessment may also have influenced the pooled results, including differences in sample storage conditions, sequencing platforms, targeted 16S rRNA regions, and bioinformatic pipelines. Most studies relied on 16S rRNA sequencing rather than shotgun meta-genomics or meta-transcriptomics, limiting taxonomic resolution and functional interpretation. Finally, important confounders such as smoking status, diet, antibiotic exposure, and comorbidities were not uniformly controlled across studies.

Future research should prioritize well-designed prospective longitudinal studies to determine whether the observed gut microbiota alterations precede lung cancer development, change with disease progression, or predict treatment outcomes. In parallel, mechanistic studies integrating metagenomics, metabolomics, and host immune profiling are needed to clarify how specific microbial taxa and microbial metabolites influence lung carcinogenesis, systemic inflammation, and response to immunotherapy through the gut-lung axis. Such work will be essential to validate the clinical significance of these microbial signatures and to determine whether they can be translated into robust biomarkers or microbiota-targeted therapeutic strategies in lung cancer.

## 5 Conclusion

In conclusion, our meta-analysis consolidates compelling evidence that gut microbiota dysbiosis is intricately associated with lung cancer. The consistent alterations in microbial diversity and taxonomic abundance patterns suggest that the gut microbiome could serve as both a biomarker and a therapeutic target in lung cancer. As our understanding of host-microbiota-tumor interactions deepens, integrative strategies that incorporate microbiota profiling into precision oncology hold promise for transforming lung cancer diagnosis and therapy.

## Data Availability

Publicly available datasets were analyzed in this study. This data can be found: PROSPERO (registration number: CRD42024537463).

## References

[ref1] Adjei A. A. (2019). Lung cancer worldwide. J. Thorac. Oncol. 14:956. doi: 10.1016/j.jtho.2019.04.001, 31122558

[ref2] Ankudavicius V. Nikitina D. Lukosevicius R. Tilinde D. Salteniene V. Poskiene L. . (2024). Detailed characterization of the lung-gut microbiome Axis reveals the link between PD-L1 and the microbiome in non-small-cell lung Cancer patients. Int. J. Mol. Sci. 25:2323. doi: 10.3390/ijms25042323, 38396998 PMC10889071

[ref3] Cassol I. Ibañez M. Bustamante J. P. (2025). Key features and guidelines for the application of microbial alpha diversity metrics. Sci. Rep. 15:622. doi: 10.1038/s41598-024-77864-y, 39753610 PMC11698868

[ref4] Chau J. Yadav M. Liu B. Furqan M. Dai Q. Shahi S. . (2021). Prospective correlation between the patient microbiome with response to and development of immune-mediated adverse effects to immunotherapy in lung cancer. BMC Cancer 21:808. doi: 10.1186/s12885-021-08530-z, 34256732 PMC8278634

[ref5] Chen S. Gui R. Zhou X. H. Zhang J. H. Jiang H. Y. Liu H. T. . (2022). Combined microbiome and metabolome analysis reveals a novel interplay between intestinal Flora and Serum metabolites in lung Cancer. Front. Cell. Infect. Microbiol. 12:885093. doi: 10.3389/fcimb.2022.885093, 35586253 PMC9108287

[ref6] Chen P. Liu Y. Wen Y. Zhou C. (2022). Non-small cell lung cancer in China. Cancer Commun. 42, 937–970. doi: 10.1002/cac2.12359, 36075878 PMC9558689

[ref7] Chen Z. Wang Z. Ma H. Bao H. Jiang T. Yang T. . (2024). Immune cells mediated the causal relationship between the gut microbiota and lung cancer: a Mendelian randomization study. Front. Microbiol. 15:1390722. doi: 10.3389/fmicb.2024.1390722, 38765682 PMC11099228

[ref8] Chen J. Yu X. Wu X. Chai K. Wang S. (2024). Causal relationships between gut microbiota, immune cell, and non-small cell lung cancer: a two-step, two-sample Mendelian randomization study. J. Cancer 15, 1890–1897. doi: 10.7150/jca.92699, 38434967 PMC10905411

[ref9] Collins S. L. Stine J. G. Bisanz J. E. Okafor C. D. Patterson A. D. (2023). Bile acids and the gut microbiota: metabolic interactions and impacts on disease. Nat. Rev. Microbiol. 21, 236–247. doi: 10.1038/s41579-022-00805-x, 36253479 PMC12536349

[ref10] Fan Y. Pedersen O. (2021). Gut microbiota in human metabolic health and disease. Nat. Rev. Microbiol. 19, 55–71. doi: 10.1038/s41579-020-0433-9, 32887946

[ref11] Fan J. Zhou Y. Meng R. Tang J. Zhu J. Aldrich M. C. . (2023). Cross-talks between gut microbiota and tobacco smoking: a two-sample Mendelian randomization study. BMC Med. 21:163. doi: 10.1186/s12916-023-02863-1, 37118782 PMC10148467

[ref12] Feng C. Li N. Gao G. He Q. Kwok L. Y. Zhang H. (2024). Dynamic changes of the gut microbiota and its functional metagenomic potential during the development of non-small cell lung Cancer. Int. J. Mol. Sci. 25:3768. doi: 10.3390/ijms25073768, 38612577 PMC11011768

[ref13] Guo Y. Yuan W. Lyu N. Pan Y. Cao X. Wang Y. . (2023). Association studies on gut and lung microbiomes in patients with lung adenocarcinoma. Microorganisms 11:546. doi: 10.3390/microorganisms11030546, 36985120 PMC10059697

[ref14] Haberman Y. Kamer I. Amir A. Goldenberg S. Efroni G. Daniel-Meshulam I. . (2023). Gut microbial signature in lung cancer patients highlights specific taxa as predictors for durable clinical benefit. Sci. Rep. 13:2007. doi: 10.1038/s41598-023-29136-4, 36737654 PMC9898251

[ref15] Hendriks L. E. Remon J. Faivre-Finn C. Garassino M. C. Heymach J. V. Kerr K. M. . (2024). Non-small-cell lung cancer. Nat. Rev. Dis. Primers 10:71. doi: 10.1038/s41572-024-00551-9 39327441

[ref16] Hsu C. L. Schnabl B. (2023). The gut–liver axis and gut microbiota in health and liver disease. Nat. Rev. Microbiol. 21, 719–733. doi: 10.1038/s41579-023-00904-3, 37316582 PMC10794111

[ref17] Huang J. Deng Y. Tin M. S. Lok V. Ngai C. H. Zhang L. . (2022). Distribution, risk factors, and temporal trends for lung cancer incidence and mortality: a global analysis. Chest 161, 1101–1111. doi: 10.1016/j.chest.2021.12.655, 35026300

[ref18] Jiang H. Zeng W. Zhang X. Li Y. Wang Y. Peng A. . (2023). Gut microbiota and its metabolites in non-small cell lung cancer and brain metastasis: from alteration to potential microbial markers and drug targets. Front. Cell. Infect. Microbiol. 13:1211855. doi: 10.3389/fcimb.2023.1211855, 38304459 PMC10830900

[ref19] Kim B.-R. Shin J. Guevarra R. B. Lee J. H. Kim D. W. Seol K.-H. . (2017). Deciphering diversity indices for a better understanding of microbial communities. J. Microbiol. Biotechnol. 27, 2089–2093. doi: 10.4014/jmb.1709.09027, 29032640

[ref20] Kulecka M. Czarnowski P. Bałabas A. Turkot M. Kruczkowska-Tarantowicz K. Żeber-Lubecka N. . (2024). Microbial and metabolic gut profiling across seven malignancies identifies fecal Faecalibacillus intestinalis and formic acid as commonly altered in Cancer patients. Int. J. Mol. Sci. 25:8026. doi: 10.3390/ijms25158026, 39125593 PMC11311272

[ref21] Leiter A. Veluswamy R. R. Wisnivesky J. P. (2023). The global burden of lung cancer: current status and future trends. Nat. Rev. Clin. Oncol. 20, 624–639. doi: 10.1038/s41571-023-00798-3, 37479810

[ref22] Li X. Shang S. Wu M. Song Q. Chen D. (2024). Gut microbial metabolites in lung cancer development and immunotherapy: novel insights into gut-lung axis. Cancer Lett. 598:217096. doi: 10.1016/j.canlet.2024.217096, 38969161

[ref23] Li Y. Wang K. Zhang Y. Yang J. Wu Y. Zhao M. (2023). Revealing a causal relationship between gut microbiota and lung cancer: a Mendelian randomization study. Front. Cell. Infect. Microbiol. 13:1200299. doi: 10.3389/fcimb.2023.1200299, 37829610 PMC10565354

[ref24] Liu F. Li J. Guan Y. Lou Y. Chen H. Xu M. . (2019). Dysbiosis of the gut microbiome is associated with tumor biomarkers in lung Cancer. Int. J. Biol. Sci. 15, 2381–2392. doi: 10.7150/ijbs.35980, 31595156 PMC6775324

[ref25] Liu N.-N. Ma Q. Ge Y. Yi C.-X. Wei L.-Q. Tan J.-C. . (2020). Microbiome dysbiosis in lung cancer: from composition to therapy. NPJ Precision Oncology 4:33. doi: 10.1038/s41698-020-00138-z, 33303906 PMC7730185

[ref26] Liu W. Pi Z. Wang X. Shang C. Song C. Wang R. . (2024). Microbiome and lung cancer: carcinogenic mechanisms, early cancer diagnosis, and promising microbial therapies. Crit. Rev. Oncol. Hematol. 196:104322. doi: 10.1016/j.critrevonc.2024.104322, 38460928

[ref27] Lu H. Gao N. L. Tong F. Wang J. Li H. Zhang R. . (2021). Alterations of the human lung and gut microbiomes in non-small cell lung carcinomas and distant metastasis. Microbiol. Spectrum 9:e0080221. doi: 10.1128/Spectrum.00802-21, 34787462 PMC8597645

[ref28] Lu X. Xiong L. Zheng X. Yu Q. Xiao Y. Xie Y. (2023). Structure of gut microbiota and characteristics of fecal metabolites in patients with lung cancer. Front. Cell. Infect. Microbiol. 13:1170326. doi: 10.3389/fcimb.2023.1170326, 37577375 PMC10415071

[ref29] Lu Y. Yuan X. Wang M. He Z. Li H. Wang J. . (2022). Gut microbiota influence immunotherapy responses: mechanisms and therapeutic strategies. J. Hematol. Oncol. 15:47. doi: 10.1186/s13045-022-01273-9, 35488243 PMC9052532

[ref30] Luan J. Zhang F. Suo L. Zhang W. Li Y. Yu X. . (2024). Analyzing lung cancer risks in patients with impaired pulmonary function through characterization of gut microbiome and metabolites. BMC Pulm. Med. 24:1. doi: 10.1186/s12890-023-02825-6, 38166904 PMC10759599

[ref31] Mager L. F. Burkhard R. Pett N. Cooke N. C. A. Brown K. Ramay H. . (2020). Microbiome-derived inosine modulates response to checkpoint inhibitor immunotherapy. Science 369, 1481–1489. doi: 10.1126/science.abc3421, 32792462

[ref32] Miyauchi E. Taida T. Uchiyama K. Nakanishi Y. Kato T. Koido S. . (2025). Smoking affects gut immune system of patients with inflammatory bowel diseases by modulating metabolomic profiles and mucosal microbiota. Gut 75, 46–56. doi: 10.1136/gutjnl-2025-334922, 40854688

[ref33] Montanari E. Bernardo G. Le Noci V. Anselmi M. Pupa S. M. Tagliabue E. . (2025). Biofilm formation by the host microbiota: a protective shield against immunity and its implication in cancer. Mol. Cancer 24, 148–116. doi: 10.1186/s12943-025-02348-0, 40399923 PMC12093748

[ref34] Mountzios G. Remon J. Hendriks L. E. García-Campelo R. Rolfo C. Van Schil P. . (2023). Immune-checkpoint inhibition for resectable non-small-cell lung cancer—opportunities and challenges. Nat. Rev. Clin. Oncol. 20, 664–677. doi: 10.1038/s41571-023-00794-7 37488229

[ref35] Nabet B. Y. Hamidi H. Lee M. C. Banchereau R. Morris S. Adler L. . (2024). Immune heterogeneity in small-cell lung cancer and vulnerability to immune checkpoint blockade. Cancer Cell 42, 429–443.e4. doi: 10.1016/j.ccell.2024.01.010, 38366589

[ref36] Ni B. Kong X. Yan Y. Fu B. Zhou F. Xu S. (2023). Combined analysis of gut microbiome and serum metabolomics reveals novel biomarkers in patients with early-stage non-small cell lung cancer. Front. Cell. Infect. Microbiol. 13:1091825. doi: 10.3389/fcimb.2023.1091825, 36743312 PMC9895385

[ref37] Page M. J. McKenzie J. E. Bossuyt P. M. Boutron I. Hoffmann T. C. Mulrow C. D. . (2021). The PRISMA 2020 statement: an updated guideline for reporting systematic reviews. BMJ 372:n71. doi: 10.1136/bmj.n71, 33782057 PMC8005924

[ref38] Qian X. Zhang H. Y. Li Q. L. Ma G. J. Chen Z. Ji X. M. . (2022). Integrated microbiome, metabolome, and proteome analysis identifies a novel interplay among commensal bacteria, metabolites and candidate targets in non-small cell lung cancer. Clin. Transl. Med. 12:e947. doi: 10.1002/ctm2.947, 35735103 PMC9218934

[ref39] Qin X. Bi L. Yang W. He Y. Gu Y. Yang Y. . (2022). Dysbiosis of the gut microbiome is associated with histopathology of lung cancer. Front. Microbiol. 13:918823. doi: 10.3389/fmicb.2022.918823 35774470 PMC9237568

[ref40] Rackaityte E. Lynch S. V. (2020). The human microbiome in the 21st century. Nat. Commun. 11:5256. doi: 10.1038/s41467-020-18983-8, 33067429 PMC7567807

[ref41] Rudin C. M. Brambilla E. Faivre-Finn C. Sage J. (2021). Small-cell lung cancer. Nat. Rev. Dis. Prim. 7:3. doi: 10.1038/s41572-020-00235-0, 33446664 PMC8177722

[ref42] Sepich-Poore G. D. Zitvogel L. Straussman R. Hasty J. Wargo J. A. Knight R. (2021). The microbiome and human cancer. Science 371:eabc4552. doi: 10.1126/science.abc4552, 33766858 PMC8767999

[ref43] Shen W. Tang D. Deng Y. Li H. Wang T. Wan P. . (2021). Association of gut microbiomes with lung and esophageal cancer: a pilot study. World J. Microbiol. Biotechnol. 37:128. doi: 10.1007/s11274-021-03086-3, 34212246

[ref44] Shen J. Wang S. Xia H. Han S. Wang Q. Wu Z. . (2023). *Akkermansia muciniphila* attenuated lipopolysaccharide-induced acute lung injury by modulating the gut microbiota and SCFAs in mice. Food Funct. 14, 10401–10417. doi: 10.1039/d3fo04051h, 37955584

[ref45] Shoji F. Minemura A. Kozuma Y. Nouno T. Takeoka H. Matsumoto A. . (2024). A prospective observational study analyzing the diversity and specific composition of the oral and gut microbiota in lung cancer patients. Anticancer Res. 44, 5067–5080. doi: 10.21873/anticanres.17331, 39477328

[ref46] Stang A. (2010). Critical evaluation of the Newcastle-Ottawa scale for the assessment of the quality of nonrandomized studies in meta-analyses. Eur. J. Epidemiol. 25, 603–605. doi: 10.1007/s10654-010-9491-z, 20652370

[ref47] Suzuki T. A. Fitzstevens J. L. Schmidt V. T. Enav H. Huus K. E. Mbong Ngwese M. . (2022). Codiversification of gut microbiota with humans. Science 377, 1328–1332. doi: 10.1126/science.abm7759, 36108023 PMC10777373

[ref48] Tesolato S. Vicente-Valor J. Paz-Cabezas M. Gómez-Garre D. Sánchez-González S. Ortega-Hernández A. . (2024). Gut microbiota signatures with potential clinical usefulness in colorectal and non-small cell lung cancers. Biomedicine 12:703. doi: 10.3390/biomedicines12030703, 38540316 PMC10967942

[ref49] Tomita Y. Sakata S. Imamura K. Iyama S. Jodai T. Saruwatari K. . (2023). Association of *Clostridium butyricum* therapy using the live bacterial product CBM588 with the survival of patients with lung cancer receiving chemoimmunotherapy combinations. Cancers (Basel) 16:47. doi: 10.3390/cancers16010047, 38201474 PMC10778075

[ref50] Verma A. Bhagchandani T. Rai A. Nikita Sardarni U. K. Bhavesh N. S. . (2024). Short-chain fatty acid (SCFA) as a connecting link between microbiota and gut-lung axis─ a potential therapeutic intervention to improve lung health. ACS Omega 9, 14648–14671. doi: 10.1021/acsomega.3c05846 38585101 PMC10993281

[ref51] Wang S. Chen H. Yang H. Zhou K. Bai F. Wu X. . (2022). Gut microbiome was highly related to the regulation of metabolism in lung adenocarcinoma patients. Front. Oncol. 12:790467. doi: 10.3389/fonc.2022.790467, 35592677 PMC9113755

[ref52] Wang T. Su W. Li L. Wu H. Huang H. Li Z. (2024). Alteration of the gut microbiota in patients with lung cancer accompanied by chronic obstructive pulmonary diseases. Heliyon 10:e30380. doi: 10.1016/j.heliyon.2024.e30380, 38737249 PMC11088322

[ref53] Wang B. Yao M. Lv L. Ling Z. Li L. (2017). The human microbiota in health and disease. Engineering 3, 71–82. doi: 10.1016/J.ENG.2017.01.008

[ref54] Wei Y. F. Huang M. S. Huang C. H. Yeh Y. T. Hung C. H. (2022). Impact of gut Dysbiosis on the risk of non-small-cell lung Cancer. Int. J. Environ. Res. Public Health 19:15991. doi: 10.3390/ijerph192315991, 36498063 PMC9740010

[ref55] Xiang Wan W.W. Liu J. Tong T. (2014). Estimating the sample mean and standard deviation from the sample size, median, range and/or interquartile range. BMC Med. Res. Methodol. 14:135. doi: 10.1186/1471-2288-14-135. 25524443 PMC4383202

[ref56] Yang Y. Du L. Shi D. Kong C. Liu J. Liu G. . (2021). Dysbiosis of human gut microbiome in young-onset colorectal cancer. Nat. Commun. 12:6757. doi: 10.1038/s41467-021-27112-y, 34799562 PMC8604900

[ref57] Yang Y. Guo Y. Ding Y. Li J. Liang L. Shi D. . (2025). Perioperative administration of CBM588 in colorectal cancer radical surgery: a single-center, randomized controlled trial. Cell Rep. Med. 6:102234. doi: 10.1016/j.xcrm.2025.102234 40633539 PMC12281423

[ref58] Yang L. Li A. Wang Y. Zhang Y. (2023). Intratumoral microbiota: roles in cancer initiation, development and therapeutic efficacy. Signal Transduct. Target. Ther. 8:35. doi: 10.1038/s41392-022-01304-4, 36646684 PMC9842669

[ref59] Zhang J. Hou L. Lei S. Li Y. Xu G. (2025). The causal relationship of cigarette smoking to metabolic disease risk and the possible mediating role of gut microbiota. Ecotoxicol. Environ. Saf. 290:117522. doi: 10.1016/j.ecoenv.2024.117522, 39709709

[ref60] Zhang W. Q. Zhao S. K. Luo J. W. Dong X. P. Hao Y. T. Li H. . (2018). Alterations of fecal bacterial communities in patients with lung cancer. Am. J. Transl. Res. 10, 3171–3185. 30416659 PMC6220220

[ref61] Zhang M. Zhou H. Xu S. Liu D. Cheng Y. Gao B. . (2020). The gut microbiome can be used to predict the gastrointestinal response and efficacy of lung cancer patients undergoing chemotherapy. Ann Palliat Med 9, 4211–4227. doi: 10.21037/apm-20-2183, 33302682

[ref62] Zhao F. An R. Wang L. Shan J. Wang X. (2021). Specific gut microbiome and serum metabolome changes in lung Cancer patients. Front. Cell. Infect. Microbiol. 11:725284. doi: 10.3389/fcimb.2021.725284, 34527604 PMC8435782

[ref63] Zhao H. Li D. Liu J. Zhou X. Han J. Wang L. . (2023). *Bifidobacterium breve* predicts the efficacy of anti-PD-1 immunotherapy combined with chemotherapy in Chinese NSCLC patients. Cancer Med. 12, 6325–6336. doi: 10.1002/cam4.5312, 36205311 PMC10028067

[ref64] Zheng Y. Fang Z. Xue Y. Zhang J. Zhu J. Gao R. . (2020). Specific gut microbiome signature predicts the early-stage lung cancer. Gut Microbes 11, 1030–1042. doi: 10.1080/19490976.2020.1737487, 32240032 PMC7524275

[ref65] Zhuang H. Cheng L. Wang Y. Zhang Y. K. Zhao M. F. Liang G. D. . (2019). Dysbiosis of the gut microbiome in lung Cancer. Front. Cell. Infect. Microbiol. 9:112. doi: 10.3389/fcimb.2019.00112, 31065547 PMC6489541

