# Supplementary material for: The composition alteration of gut microbiota in lung cancer: a systematic review and meta-analysis
Source: Front Microbiol. 2026 Jul 20;17:1873706. doi: 10.3389/fmicb.2026.1873706 (PMC13429741; doi:10.3389/fmicb.2026.1873706)
Supplement: Supplementary file 1 [file Data_Sheet_1.PDF]

Supplementary Material

The Composition Alteration of Gut Microbiota in Lung Cancer: A Systematic Review and Meta-Analysis

Xiaona Xu[1*], Ting Yu[1*], Hongying Wu[2*], Yang Guo[3], Meng Li[4], Yaozu Han[5#], Lei Zhao[6#], Xinjuan Yu[5#]

Content:

- **Supplementary Table 1. Equations used to search for articles within databases.**
- **Supplementary Table 2. Newcastle-Ottawa scale showing quality evaluation of studies included.**
- **Supplementary Table 3. Receiver operating characteristic (ROC) analysis was conducted on microbiota markers to distinguish lung cancer patients from controls in the included studies.**
- **Supplementary Table 4. Egger's Test Results for α-Diversity Indices.**
- **Supplementary Figure 1. Sensitivity analysis assessing heterogeneity and publication bias.**
- **Supplementary Figure 2. Funnel plots assessing publication bias.**

Supplementary Table 1. Equations used to search for articles within databases.

Databases	Equations used
Pubmed	(((((microbiota[MeSH Terms]) OR (microbiota[Title/Abstract] OR microbiome*[Title/Abstract] OR microbiota*[Title/Abstract] OR microbial communit*[Title/Abstract])) OR (flore*[Title/Abstract] OR flora[Title/Abstract] OR microflor*[Title/Abstract])) OR ((metagenome[MeSH Terms]) OR (metagenome[Title/Abstract] OR metagenomes[Title/Abstract]))) OR ((dysbiosis[MeSH Terms]) OR (dysbiosis[Title/Abstract] OR dysbioses[Title/Abstract] OR disbiosis[Title/Abstract] OR disbioses[Title/Abstract] OR dysbacterios*[Title/Abstract] OR disbacterios*[Title/Abstract] OR bacterial[Title/Abstract]))) AND ((lung neoplasms[MeSH Terms]) OR (lung cancer*[Title/Abstract] OR lung neoplasms[Title/Abstract] OR lung neoplasm[Title/Abstract] OR pulmonary neoplasm*[Title/Abstract] OR pulmonary cancer*[Title/Abstract] OR Cancer of the Lung[Title/Abstract] OR Cancer of Lung[Title/Abstract]))
Web of Science	#1 (((TS=(microbiota OR microbiome* OR microbiota* OR microbial communit*)) OR TS=(flore* OR flora OR microflor*)) OR TS=(metagenome OR metagenomes)) OR TS=(dysbiosis OR dysbioses OR disbiosis OR disbioses OR dysbacterios* OR disbacterios* OR bacterial)<br>#2 TS=(lung cancer* OR lung neoplasms OR lung neoplasm OR pulmonary neoplasm* OR pulmonary cancer* OR Cancer of the Lung OR Cancer of Lung)<br>#3 (#1) AND #2
cochrane library	#1 microbiota[MeSH Terms]<br>#2 microbiota OR microbiome* OR microbiota* OR microbial communit*<br>#3 flore* OR flora OR microflor*<br>#4 #1 OR #2 OR #3<br>#5 metagenome[MeSH Terms]<br>#6 metagenome OR metagenomes<br>#7 #5 OR #6<br>#8 dysbiosis[MeSH Terms]<br>#9 dysbiosis OR dysbioses OR disbiosis OR disbioses OR dysbacterios* OR disbacterios* OR bacterial<br>#10 #8 OR #9<br>#11 #4 OR #7 OR #10<br>#12 lung neoplasms[MeSH Terms]<br>#13 lung cancer* OR lung neoplasms OR lung neoplasm OR pulmonary neoplasm* OR pulmonary cancer* OR Cancer of the Lung OR Cancer of Lung<br>#14 #12 OR #13<br>#15 #11 AND #14
Embase	Query('microbiota'/exp OR microbiota:ti,ab,kw OR microbiome*:ti,ab,kw OR microbiota*:ti,ab,kw OR 'microbial communit*':ti,ab,kw OR flore*:ti,ab,kw OR flora:ti,ab,kw OR microflor*:ti,ab,kw OR 'metagenome'/exp OR metagenome:ti,ab,kw OR metagenomes:ti,ab,kw OR

	'dysbiosis'/exp OR dysbiosis:ti,ab,kw OR dysbioses:ti,ab,kw OR disbiosis:ti,ab,kw OR disbioses:ti,ab,kw OR dysbacterios*:ti,ab,kw OR disbacterios*:ti,ab,kw OR bacterial:ti,ab,kw) AND ('lung neoplasms'/exp OR 'lung cancer*':ti,ab,kw OR 'lung neoplasms':ti,ab,kw OR 'lung neoplasm':ti,ab,kw OR 'pulmonary neoplasm*':ti,ab,kw OR 'pulmonary cancer*':ti,ab,kw OR 'cancer of the lung':ti,ab,kw OR 'cancer of lung':ti,ab,kw)

Supplementary Table 2. Newcastle-Ottawa scale showing quality evaluation of studies included.

Publications	Selection				Comparability	Exposure			Overall Score (Max. of 10)	Risk of Bias
	Definition of cases	Representativeness of the cases	Selection of Controls	Definition of Controls	Comparability of cases and controls	Ascertainment of exposure	Same method of ascertainment for cases and controls	Non-Response rate		
Ankudavicius et al. 2024(Ankudavicius et al., 2024)	1	1	1	1	0	1	1	0	6	Moderate
Chau et al. 2021(Chau et al., 2021)	1	1	1	1	0	1	1	0	6	Moderate
Chen et al. 2022(Chen et al., 2022)	1	1	1	1	1	1	1	0	7	High
Feng et al. 2024(Feng et al., 2024)	1	1	1	1	0	1	1	0	6	Moderate
Guo et al. 2023(Guo et al., 2023)	1	1	2	1	1	1	1	0	8	High
Haberman et al. 2023(Haberman et al., 2023)	1	1	1	1	2	1	1	0	8	High
Jiang et al. 2023(Jiang et al., 2023)	1	1	1	1	2	1	1	0	8	High
Kulecka et al. 2024(Kulecka et al., 2024)	1	1	1	1	1	1	1	0	7	High

Liu et al. 2019(Liu et al., 2019)	1	1	1	1	2	1	1	0	8	High
Lu et al. 2021(Lu et al., 2021)	1	1	2	1	2	1	1	0	9	High
Lu et al. 2023(Lu et al., 2023)	1	1	1	1	2	1	1	0	8	High
Luan et al. 2024(Luan et al., 2024)	1	1	1	1	2	1	1	0	8	High
Ni et al. 2023(Ni et al., 2023)	1	1	2	1	2	1	1	0	9	High
Qian et al. 2022(Qian et al., 2022)	1	1	1	1	2	1	1	0	8	High
Qin et al. 2022(Qin et al., 2022)	1	1	1	1	2	1	1	0	8	High
Shen et al. 2021(Shen et al., 2021)	1	1	1	1	1	1	1	0	7	High
Shoji et al. 2024(Shoji et al., 2024)	1	1	1	1	2	1	1	0	8	High
Tesolato et al. 2024(Tesolato et al., 2024)	1	1	1	1	0	1	1	0	6	Moderate
Wang et al. 2022(Wang et al., 2022)	1	1	1	1	2	1	1	0	8	High
Wang et al. 2024(Wang et al., 2024)	1	1	1	1	2	1	1	0	8	High

Wei et al. 2022(Wei et al., 2022)	1	1	1	1	2	1	1	0	8	High
Zhang et al. 2018(Zhang et al., 2018)	1	1	1	1	1	1	1	0	7	High
Zhang et al. 2020(Zhang et al., 2020)	1	1	1	1	0	1	1	0	6	Moderate
Zhao et al. 2021(Zhao et al., 2021)	1	1	1	1	2	1	1	0	8	High
Zhao et al. 2023(Zhao et al., 2023)	1	1	1	1	1	1	1	0	7	High
Zheng et al. 2020(Zheng et al., 2020)	1	1	1	1	2	1	1	0	8	High
Zhuang et al. 2019(Zhuang et al., 2019)	1	1	1	1	1	1	1	0	7	High

The NOS scale has a maximum score of 10, Case-control studies with an overall score of ≥7 were considered high quality, those scoring 4–6 were classified as moderate quality, and those scoring <4 were regarded as low quality.

Supplementary Table 3. Receiver operating characteristic (ROC) analysis was conducted on microbiota markers to distinguish lung cancer patients from controls in the included studies.

Study	Marker	AUC (95%CI)	Sensitivity (%)	Specificity (%)
Jiang et al. 2023(Jiang et al., 2023)	6 genera (*Faecalibacterium, Klesiella, Butyricicoccus, Bifidobacterium, Streptococcus, Blautia*)	0.884 (NR)	NR	NR
	Faecalibacterium	0.732 (NR)	NR	NR
	Klesiella	0.755 (NR)	NR	NR
	Butyricicoccus	0.709 (NR)	NR	NR
	Bifidobacterium	0.731 (NR)	NR	NR
	Streptococcus	0.675 (NR)	NR	NR
	Blautia	0.639 (NR)	NR	NR
Shen et al. 2021(Shen et al., 2021)	*Enterococcus (relative abundance)*	0.844 (0.729–0.959)	0.941	0.649
	Enterococcus (absolute abundance)	0.838 (0.726–0.95)	0.882	0.730
	Streptococcus (relative abundance)	0.935 (0.844–1.000)	0.882	0.919
	Streptococcus (absolute abundance)	0.793 (0.638–0.949)	0.706	0.919
	Klebsiella (relative abundance)	0.957 (0.895–1.000)	0.882	0.946
	five indicators above mixed	0.959 (0.899–1.000)	0.882	1.000
	Klebsiella and Streptococcus (relative abundance)	0.946 (0.880–1.000)	0.882	0.919
Wang et al. 2022(Wang et al., 2022)	*Bacteroides*	0.767 (NR)	NR	NR
	Pseudomonas	0.781 (NR)	NR	NR
	Ruminococcus gnavus group	0.746 (NR)	NR	NR
Wei et al. 2022(Wei et al., 2022)	*Bacteroides caccae*	0.669 (NR)	NR	NR
	Anaerotruncus spp	0.714 (NR)	NR	NR
	Bacteroides caccae and Anaerotruncus spp	0.676 (NR)	NR	NR
Tesolato et al. 2024(Tesolato et al., 2024)	*DTU089 and Ruminococcaceae Incertae Sedis*	0.747 (NR)	0.737	0.750

Supplementary Table 4. Egger's Test Results for α Diversity Indices.

α diversity index	Types of Effect Values	Coefficient	*P* value	95% confidence interval
ACE index	slope	1.03	0.29	[-1.23, 3.29]
	bias	-6.18	0.14	[-15.20, 2.84]
CHAO 1 index	slope	-0.23	0.74	[-1.71, 1.24]
	bias	-0.30	0.91	[-6.07, 5.47]
observed species	slope	0.25	0.75	[-1.44, 1.95]
	bias	-1.99	0.54	[-9.02, 5.04]
Pielou's evenness	slope	-0.19	0.80	[-2.29, 1.92]
	bias	0.60	0.85	[-8.93, 10.14]
Shannon index	slope	0.01	0.98	[-0.81, 0.83]
	bias	-1.06	0.52	[-4.40, 2.28]
Simpson index	slope	0.33	0.57	[-0.90, 1.57]
	bias	-1.74	0.45	[-6.51, 3.04]

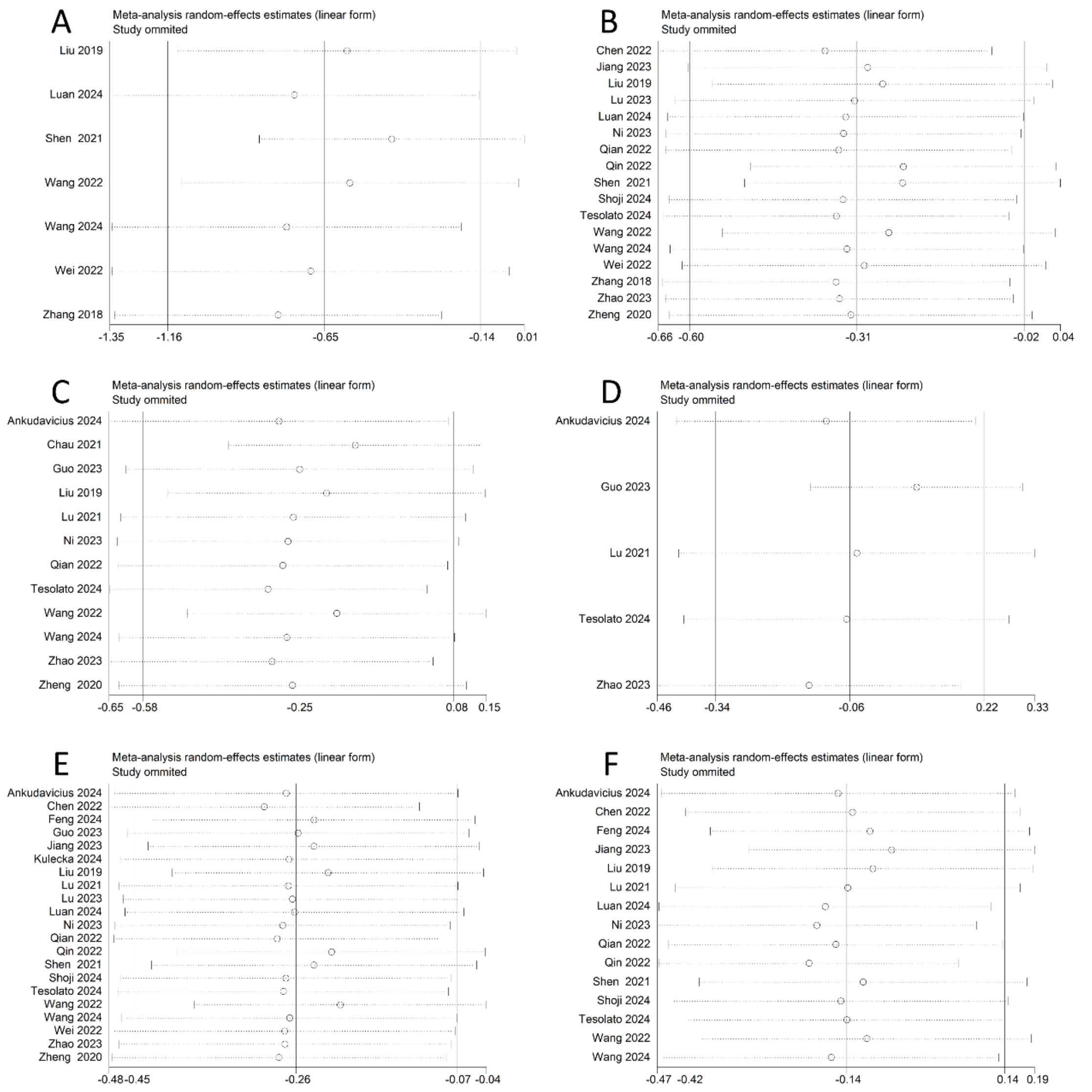

Supplementary Figure 1. Sensitivity analysis assessing heterogeneity and publication bias. (A) ACE index; (B) Chao 1 index; (C) Observed species index; (D)Shannon index; (E) Simpson index.

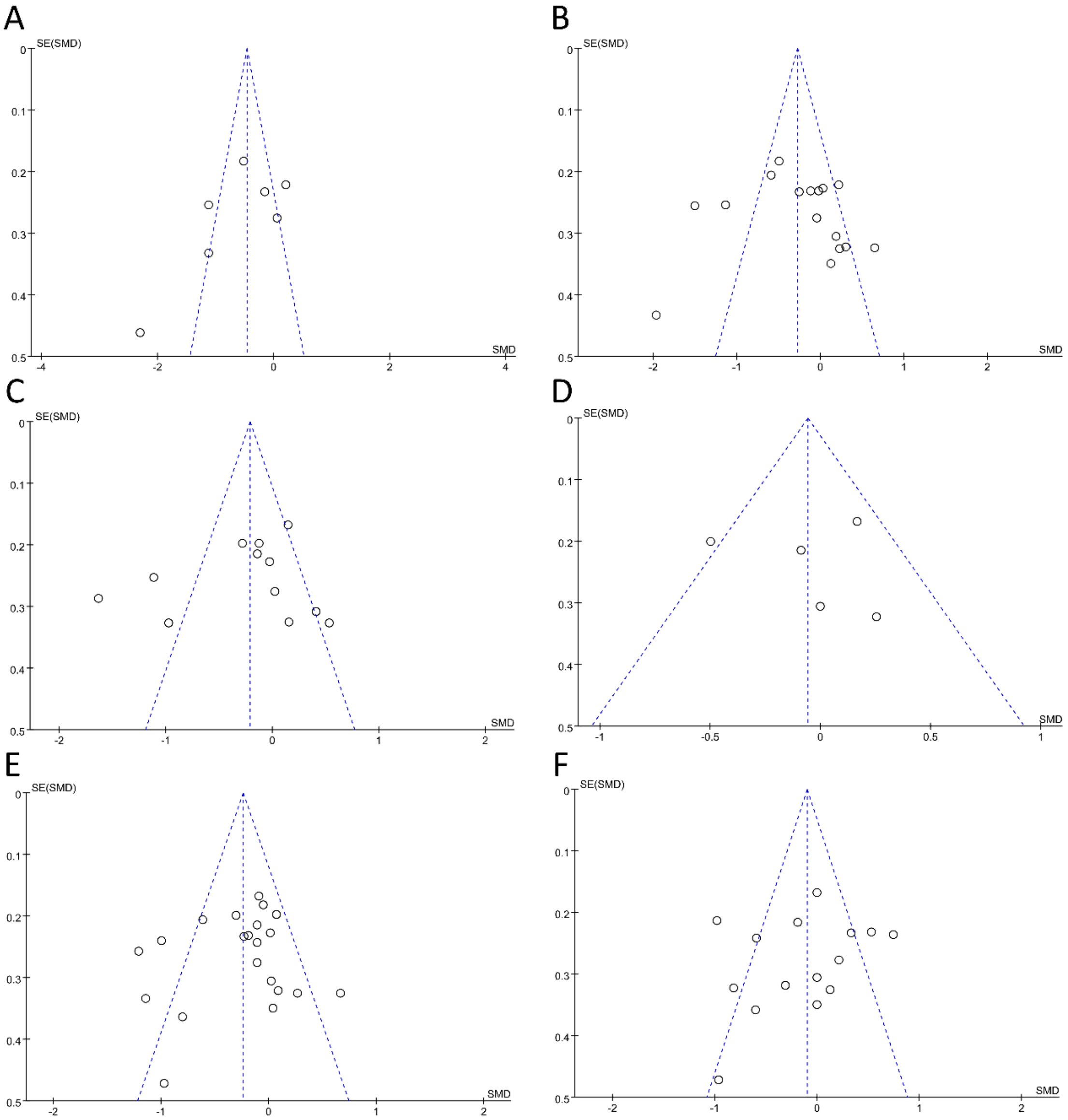

Supplementary Figure 2. Funnel plots assessing publication bias. (A) ACE index; (B) Chao 1 index; (C) Observed species index; (D)Shannon index; (E) Simpson index.

References

Ankudavicius, V., Nikitina, D., Lukosevicius, R., Tilinde, D., Salteniene, V., Poskiene, L., et al. (2024). Detailed Characterization of the Lung-Gut Microbiome Axis Reveals the Link between PD-L1 and the Microbiome in Non-Small-Cell Lung Cancer Patients. *Int J Mol Sci* 25(4). doi: 10.3390/ijms25042323.

Chau, J., Yadav, M., Liu, B., Furqan, M., Dai, Q., Shahi, S., et al. (2021). Prospective correlation between the patient microbiome with response to and development of immune-mediated adverse effects to immunotherapy in lung cancer. *BMC Cancer* 21(1)**,** 808. doi: 10.1186/s12885-021-08530-z.

Chen, S., Gui, R., Zhou, X.H., Zhang, J.H., Jiang, H.Y., Liu, H.T., et al. (2022). Combined Microbiome and Metabolome Analysis Reveals a Novel Interplay Between Intestinal Flora and Serum Metabolites in Lung Cancer. *Front Cell Infect Microbiol* 12**,** 885093. doi: 10.3389/fcimb.2022.885093.

Feng, C., Li, N., Gao, G., He, Q., Kwok, L.Y., and Zhang, H. (2024). Dynamic Changes of the Gut Microbiota and Its Functional Metagenomic Potential during the Development of Non-Small Cell Lung Cancer. *Int J Mol Sci* 25(7). doi: 10.3390/ijms25073768.

Guo, Y., Yuan, W., Lyu, N., Pan, Y., Cao, X., Wang, Y., et al. (2023). Association Studies on Gut and Lung Microbiomes in Patients with Lung Adenocarcinoma. *Microorganisms* 11(3). doi: 10.3390/microorganisms11030546.

Haberman, Y., Kamer, I., Amir, A., Goldenberg, S., Efroni, G., Daniel-Meshulam, I., et al. (2023). Gut microbial signature in lung cancer patients highlights specific taxa as predictors for durable clinical benefit. *Sci Rep* 13(1)**,** 2007. doi: 10.1038/s41598-023-29136-4.

Jiang, H., Zeng, W., Zhang, X., Li, Y., Wang, Y., Peng, A., et al. (2023). Gut microbiota and its metabolites in non-small cell lung cancer and brain metastasis: from alteration to potential microbial markers and drug targets. *Front Cell Infect Microbiol* 13**,** 1211855. doi: 10.3389/fcimb.2023.1211855.

Kulecka, M., Czarnowski, P., Bałabas, A., Turkot, M., Kruczkowska-Tarantowicz, K., Żeber-Lubecka, N., et al. (2024). Microbial and Metabolic Gut Profiling across Seven Malignancies Identifies Fecal Faecalibacillus intestinalis and Formic Acid as Commonly Altered in Cancer Patients. *Int J Mol Sci* 25(15). doi: 10.3390/ijms25158026.

Liu, F., Li, J., Guan, Y., Lou, Y., Chen, H., Xu, M., et al. (2019). Dysbiosis of the Gut Microbiome is associated with Tumor Biomarkers in Lung Cancer. *Int J Biol Sci* 15(11)**,** 2381-2392. doi: 10.7150/ijbs.35980.

Lu, H., Gao, N.L., Tong, F., Wang, J., Li, H., Zhang, R., et al. (2021). Alterations of the Human Lung and Gut Microbiomes in Non-Small Cell Lung Carcinomas and Distant Metastasis. *Microbiol Spectr* 9(3)**,** e0080221. doi: 10.1128/Spectrum.00802-21.

Lu, X., Xiong, L., Zheng, X., Yu, Q., Xiao, Y., and Xie, Y. (2023). Structure of gut microbiota and characteristics of fecal metabolites in patients with lung cancer. *Front Cell Infect Microbiol* 13**,** 1170326. doi: 10.3389/fcimb.2023.1170326.

Luan, J., Zhang, F., Suo, L., Zhang, W., Li, Y., Yu, X., et al. (2024). Analyzing lung cancer risks in patients with impaired pulmonary function through characterization of gut microbiome and metabolites. *BMC Pulm Med* 24(1)**,** 1. doi: 10.1186/s12890-023-02825-6.

Ni, B., Kong, X., Yan, Y., Fu, B., Zhou, F., and Xu, S. (2023). Combined analysis of gut microbiome and serum metabolomics reveals novel biomarkers in patients with early-stage non-small cell lung

cancer. *Front Cell Infect Microbiol* 13, 1091825. doi: 10.3389/fcimb.2023.1091825.

Qian, X., Zhang, H.Y., Li, Q.L., Ma, G.J., Chen, Z., Ji, X.M., et al. (2022). Integrated microbiome, metabolome, and proteome analysis identifies a novel interplay among commensal bacteria, metabolites and candidate targets in non-small cell lung cancer. *Clin Transl Med* 12(6), e947. doi: 10.1002/ctm2.947.

Qin, X., Bi, L., Yang, W., He, Y., Gu, Y., Yang, Y., et al. (2022). Dysbiosis of the Gut Microbiome Is Associated With Histopathology of Lung Cancer. *Front Microbiol* 13, 918823. doi: 10.3389/fmicb.2022.918823.

Shen, W., Tang, D., Deng, Y., Li, H., Wang, T., Wan, P., et al. (2021). Association of gut microbiomes with lung and esophageal cancer: a pilot study. *World J Microbiol Biotechnol* 37(8), 128. doi: 10.1007/s11274-021-03086-3.

Shoji, F., Minemura, A., Kozuma, Y., Nouno, T., Takeoka, H., Matsumoto, A., et al. (2024). A Prospective Observational Study Analyzing the Diversity and Specific Composition of the Oral and Gut Microbiota in Lung Cancer Patients. *Anticancer Res* 44(11), 5067-5080. doi: 10.21873/anticanres.17331.

Tesolato, S., Vicente-Valor, J., Paz-Cabezas, M., Gómez-Garre, D., Sánchez-González, S., Ortega-Hernández, A., et al. (2024). Gut Microbiota Signatures with Potential Clinical Usefulness in Colorectal and Non-Small Cell Lung Cancers. *Biomedicines* 12(3). doi: 10.3390/biomedicines12030703.

Wang, S., Chen, H., Yang, H., Zhou, K., Bai, F., Wu, X., et al. (2022). Gut Microbiome Was Highly Related to the Regulation of Metabolism in Lung Adenocarcinoma Patients. *Front Oncol* 12, 790467. doi: 10.3389/fonc.2022.790467.

Wang, T., Su, W., Li, L., Wu, H., Huang, H., and Li, Z. (2024). Alteration of the gut microbiota in patients with lung cancer accompanied by chronic obstructive pulmonary diseases. *Heliyon* 10(9), e30380. doi: 10.1016/j.heliyon.2024.e30380.

Wei, Y.F., Huang, M.S., Huang, C.H., Yeh, Y.T., and Hung, C.H. (2022). Impact of Gut Dysbiosis on the Risk of Non-Small-Cell Lung Cancer. *Int J Environ Res Public Health* 19(23). doi: 10.3390/ijerph192315991.

Zhang, M., Zhou, H., Xu, S., Liu, D., Cheng, Y., Gao, B., et al. (2020). The gut microbiome can be used to predict the gastrointestinal response and efficacy of lung cancer patients undergoing chemotherapy. *Ann Palliat Med* 9(6), 4211-4227. doi: 10.21037/apm-20-2183.

Zhang, W.Q., Zhao, S.K., Luo, J.W., Dong, X.P., Hao, Y.T., Li, H., et al. (2018). Alterations of fecal bacterial communities in patients with lung cancer. *Am J Transl Res* 10(10), 3171-3185.

Zhao, F., An, R., Wang, L., Shan, J., and Wang, X. (2021). Specific Gut Microbiome and Serum Metabolome Changes in Lung Cancer Patients. *Front Cell Infect Microbiol* 11, 725284. doi: 10.3389/fcimb.2021.725284.

Zhao, H., Li, D., Liu, J., Zhou, X., Han, J., Wang, L., et al. (2023). Bifidobacterium breve predicts the efficacy of anti-PD-1 immunotherapy combined with chemotherapy in Chinese NSCLC patients. *Cancer Med* 12(5), 6325-6336. doi: 10.1002/cam4.5312.

Zheng, Y., Fang, Z., Xue, Y., Zhang, J., Zhu, J., Gao, R., et al. (2020). Specific gut microbiome signature predicts the early-stage lung cancer. *Gut Microbes* 11(4), 1030-1042. doi: 10.1080/19490976.2020.1737487.

Zhuang, H., Cheng, L., Wang, Y., Zhang, Y.K., Zhao, M.F., Liang, G.D., et al. (2019). Dysbiosis of the Gut Microbiome in Lung Cancer. *Front Cell Infect Microbiol* 9, 112. doi:

10.3389/fcimb.2019.00112.
